# Human placental proteomics and exon variant studies link AAT/*SERPINA1* with spontaneous preterm birth

**DOI:** 10.1186/s12916-022-02339-8

**Published:** 2022-04-28

**Authors:** Heli Tiensuu, Antti M. Haapalainen, Pinja Tissarinen, Anu Pasanen, Tomi A. Määttä, Johanna M. Huusko, Steffen Ohlmeier, Ulrich Bergmann, Marja Ojaniemi, Louis J. Muglia, Mikko Hallman, Mika Rämet

**Affiliations:** 1 PEDEGO Research Unit and Medical Research Center Oulu, University of Oulu, PO Box 5000, 90014 Oulu, Finland; 2 Department of Children and Adolescents, Oulu University Hospital, 90014 Oulu, Finland; 3 Division of Human Genetics, Center for Prevention of Preterm Birth, Perinatal Institute, Cincinnati Children’s Hospital Medical Center, Department of Pediatrics, University of Cincinnati College of Medicine, March of Dimes Prematurity Research Center Ohio Collaborative, Cincinnati, OH 45267 USA; 4 Proteomics and Mass Spectrometry Core Facilities, Biocenter Oulu, Faculty of Biochemistry and Molecular Medicine, University of Oulu, 90014 Oulu, Finland; 5 Burroughs Wellcome Fund, Research Triangle Park, North Carolina 27709 USA; 6 Faculty of Medicine and Health Technology, Tampere University, 33014 Tampere, Finland

**Keywords:** Alpha-1-antitrypsin, Placenta, Proteomics, Preterm birth, *SERPINA1*, Trophoblast, Whole exome sequencing

## Abstract

**Background:**

Preterm birth is defined as live birth before 37 completed weeks of pregnancy, and it is a major problem worldwide. The molecular mechanisms that lead to onset of spontaneous preterm birth are incompletely understood. Prediction and evaluation of the risk of preterm birth is challenging as there is a lack of accurate biomarkers. In this study, our aim was to identify placental proteins that associate with spontaneous preterm birth.

**Methods:**

We analyzed the proteomes from placentas to identify proteins that associate with both gestational age and spontaneous labor. Next, rare and potentially damaging gene variants of the identified protein candidates were sought for from our whole exome sequencing data. Further experiments we performed on placental samples and placenta-associated cells to explore the location and function of the spontaneous preterm labor-associated proteins in placentas.

**Results:**

Exome sequencing data revealed rare damaging variants in *SERPINA1* in families with recurrent spontaneous preterm deliveries. Protein and mRNA levels of alpha-1 antitrypsin/*SERPINA1* from the maternal side of the placenta were downregulated in spontaneous preterm births. Alpha-1 antitrypsin was expressed by villous trophoblasts in the placenta, and immunoelectron microscopy showed localization in decidual fibrinoid deposits in association with specific extracellular proteins. siRNA knockdown in trophoblast-derived HTR8/SVneo cells revealed that *SERPINA1* had a marked effect on regulation of the actin cytoskeleton pathway, Slit–Robo signaling, and extracellular matrix organization.

**Conclusions:**

Alpha-1 antitrypsin is a protease inhibitor. We propose that loss of the protease inhibition effects of alpha-1 antitrypsin renders structures critical to maintaining pregnancy susceptible to proteases and inflammatory activation. This may lead to spontaneous premature birth.

**Supplementary Information:**

The online version contains supplementary material available at 10.1186/s12916-022-02339-8.

## Background

Preterm birth (PTB) is defined as live birth before 37 completed weeks of gestation [1]. Currently, we have an incomplete understanding of the factors that lead to PTB. Both maternal and fetal genomes are likely involved in the timing of birth, as well as in the onset of spontaneous PTB (SPTB) [2–4]. Several pathological processes that involve inflammation, infection, endocrine events, disorders in blood clotting, or uterine distention may activate labor before term [5].

It is challenging to evaluate and predict the risk of preterm delivery because there is a lack of accurate biomarkers and an incomplete knowledge of the pathophysiology of SPTB [6]. Several biological specimens (e.g., cervical fluid, amniotic fluid, and maternal blood) have been studied to identify SPTB-associated risk factors and pathways and biomarkers associated with adverse pregnancy outcomes [1, 6–9]. Elevated levels of fetal fibronectin (fFN) measured from cervicovaginal fluids may predict a higher risk of preterm delivery [7, 10, 11]. However, the results are inconsistent and suggest that the predictive power of fFN has somewhat low sensitivity and specificity [7, 12]. Activation of inflammatory pathways and the complement system are proposed to be involved in the pathogenesis of SPTB [6–8, 13]. Interleukins, such as IL-6 and IL-8, have been observed to be elevated in the sera or cervicovaginal fluids of mothers who gave birth preterm [7, 13], but their predictive power is limited. Despite the research described above, currently there is no efficient and reliable predictive test for preterm birth [6–8].

The placenta is a multifunctional organ involved in fetal oxygen and nutrient uptake, as well as metabolism, immune defense function, and the elimination of toxic products [14]. In addition, placental components, compartments, and fetal membranes may be involved in regulation of the duration of pregnancy and onset of human labor [15]. Proteomic analyses of human placenta have mostly focused on pathological conditions such as preeclampsia [16] and fetal growth restriction [17]. Few placental proteomic studies have focused on term labor [18] and none have focused on preterm labor. According to a study by Heywood et al., the proteome varies among different parts of the placenta [19]. The fetal side of the placenta expresses proteins in pathways associated with actin arrangement, contraction, implantation, and negative regulation of peptidases, while the sub-proteome on the maternal side is more involved in antigen presentation, energy metabolism, and protein folding [19]. Another study compared proteome differences in the human placenta between the first and third trimesters and identified 11 differentially expressed proteins [20]. Levels of four proteins—protein disulfide isomerase, tropomyosin 4 isoform 2, enolase 1, and heat shock protein A5—decreased toward term, and seven proteins—actin γ1 propeptide, heat shock protein gp96, alpha-1 antitrypsin, EF-hand domain family member D1, tubulin α1, glutathione S-transferase, and vitamin D-binding protein—increased from the first trimester to the third trimester [20].

Abnormal levels of various SERPINs are associated with pregnancy complications [21–24]. The human genome contains 36 serine protease inhibitor (SERPIN)–coding genes, which are divided into nine clades (A–I) [25]. SERPINs regulate various pathways such as blood coagulation, fibrinolysis, and inflammation [26]. Clade A (SERPINA) is the largest group, and members of clade A are antitrypsin-like extracellular proteins [25]. *SERPINA1* encodes alpha-1 antitrypsin (AAT), a 52-kDa single-chain glycoprotein [27] involved in inhibition of serine proteases such as neutrophil elastase, trypsin, chymotrypsin, and plasminogen activator [28, 29]. Neutrophil elastase in cervical secretions has been proposed to predict premature delivery [30]. In addition to protease inhibition, SERPINA1 family members prevent collagen breakdown, which triggers hypoxia [31]. Low serum levels of AAT are associated with spontaneous abortion and elevated proinflammatory cytokines [21], and SERPINA3 is a potential preeclampsia marker [22]. Additionally, higher maternal serum levels of SERPINB7 are associated with SPTB [23]. Moreover, SERPINE2 is found in villous and extravillous trophoblasts of the human placenta and affects trophoblast migration and invasion [24]. It has also been shown that a functional SNP in the promoter of the *SERPINH1* gene significantly reduced promoter activity in amnion fibroblast cells and thus increases risk of preterm premature rupture of membranes (PPROM), the leading cause of preterm birth [32].

The initial aim of this study was to identify placental proteins that may be associated with SPTB onset. We used a hypothesis-free approach by comparing the proteomes of human placentas from SPTB, spontaneous term birth (STB), and elective preterm birth (EPTB). After we identified protein candidates, we investigated the whole exon sequences from mothers and children of families with more than one SPTB. Our aim was to identify rare, likely damaging variants that lead to SPTB. This yielded a single candidate protein, AAT, which is encoded by *SERPINA1*. To explore the function of AAT/*SERPINA1* in the placentas of SPTBs, we performed further experiments on placental samples and placenta-derived cells. We propose that AAT/*SERPINA1* is an important resistance factor in the inflammatory, endocrine, hematologic, and proteolytic events associated with predisposition to SPTB.

## Methods

### Study workflow

**Fig. 1:**
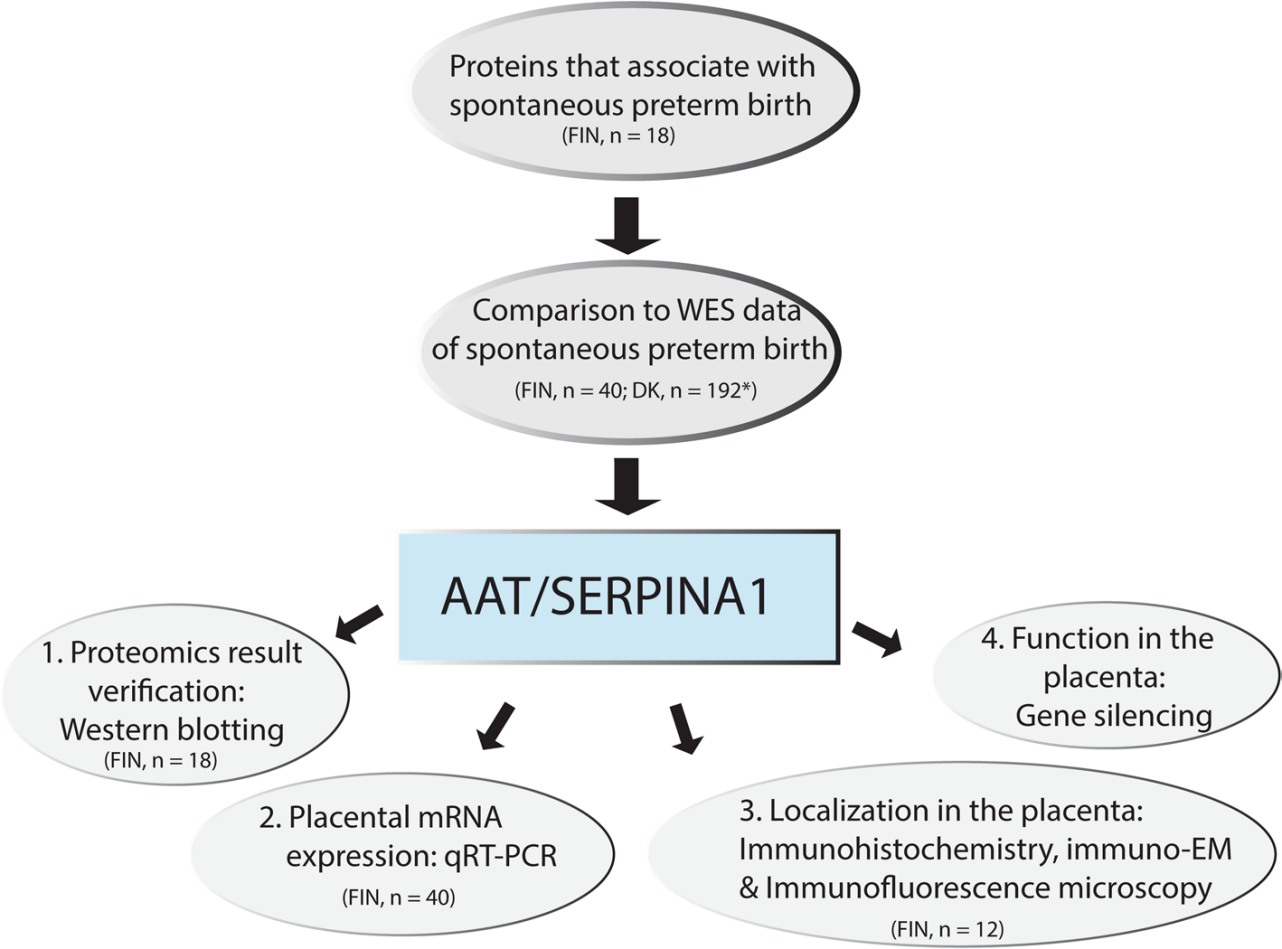
Workflow of the present study. The starting point of this study was to determine proteomes from spontaneous preterm (SPTB), spontaneous term, and elective preterm human placentas to identify proteins potentially in association with SPTB. When we compared the proteomic findings with whole exome sequencing (WES) data from SPTB families, AAT/*SERPINA1* emerged as a candidate. To verify our findings and to explore the location and function of AAT/*SERPINA1* in placentas of SPTB, we performed experiments on placental samples and placenta-associated cells. The placenta samples on which the proteomics were performed were also included in the following analyses. Only in immuno-EM and immunofluorescence microscopy, the placenta samples were different. N is number of samples, FIN is Finland, and DK is Denmark. *Finnish WES; 17 mothers and their 23 children. Danish WES; *n* = 93 sister pairs and two families with three affected siblings

Protein levels in the placenta vary during pregnancy, and some changes are associated with adverse pregnancy outcomes [5, 33]. To investigate placental proteins that are up- or downregulated during spontaneous preterm labor, we determined the proteomes of human placentas (Additional file: Fig S1) and then analyzed them with respect to spontaneous preterm birth (SPTB), elective preterm birth (EPTB), and spontaneous term birth (STB) cases. Maternal (basal plate) and fetal (chorionic plate) sides of the placenta were studied separately. SPTB and STB placentas were compared to identify protein candidates associated with gestational age, and SPTB and EPTB placentas were compared to identify proteins differently expressed in spontaneous delivery. We then compared the proteomes (SPTB vs. STB compared with SPTB vs. EPTB) to discover protein candidates associated with both short gestational age and spontaneous delivery; i.e., spontaneous preterm birth. The study workflow is presented in Fig. 1.

### Placental proteomics

We used proteomics data to explore protein levels in the human placenta. Proteomics data from placental samples were generated by two-dimensional minimal-difference gel electrophoresis and mass spectrometry, as described previously [18] (Additional file: Fig S1, Tables S1–S3). In short, placental samples were obtained from SPTB (GA 25–35 weeks, *n* = 6), EPTB (GA 25–30 weeks, *n* = 6), and STB (GA 39–41 weeks, *n* = 6) cases. Tissue biopsies were collected from both basal and chorionic plates. In the comparison, we used a *p* value of < 0.05 (Student’s *t* test) and a 1.5-fold cut-off ratio to consider if differences in protein spots were significant and selected for further identification with mass spectrometry. We excluded samples from cases that involved induction of labor, placental abnormalities, congenital malformations, preexisting medical conditions, and multiple births.

### WES data

Proteomic findings were compared with three population sets with available whole exome sequencing (WES) data. Two datasets consisted of Northern Finnish mothers with preterm deliveries (*n =* 17) [34] and their children (*n =* 23) who were born preterm (GA < 36 weeks) [35]. The third dataset was Danish WES data of European ancestry, which included 192 women from 95 families. Selection criteria for the WES analyses have been previously described in detail [36, 37], and WES was performed as previously described in detail [34]. When we compared the proteomic findings with the WES datasets, our selection criteria were rare (MAF < 1%) or common (MAF < 10%) potentially damaging variants (categories 1–3, described previously [34]) found in at least two different families.

### Western blotting of AAT

The proteomic result of AAT (P01009) was validated with quantitative western blot methodology, as described previously [18]. Tubulin α-1B was used as a reference protein to which all samples were normalized. We used mouse monoclonal anti-human AAT antibody (MA5-15521, 1:2000 dilution, Invitrogen) and rabbit monoclonal anti-human tubulin α-1B antibody (NB110-57609, 1:5000, Novusbio) to detect AAT and tubulin α-1B, respectively (Additional file: Fig S2). Quantitative western blot results were analyzed with SPSS Statistics 20.0 (IBM Corporation). The nonparametric Mann–Whitney *U* test was used to evaluate the level of statistical difference.

### qRT-PCR of *SERPINA1*

To determine mRNA levels of *SERPINA1* in the placenta, we conducted qRT-PCR. We included samples from the basal plate of the placenta and compared samples from SPTB (*n* = 18) and STB (*n* = 22). Samples were collected in 2010–2016 as described previously [38]. Inclusion criteria for gestational age were from 25 weeks to 36 weeks + 6 days for SPTB samples and from 39 weeks to 41 weeks + 6 days for STB samples.

Placental RNA was isolated as described previously [38]. The quality and quantity of isolated RNA was determined with a NanoDrop (Thermo Fisher) by measuring absorbance values at 230, 260, and 280 nm. Total RNA was converted into cDNA with the Transcriptor First Strand cDNA Synthesis Kit (Roche Diagnostics) according to the standard qRT-PCR procedure.

A LightCycler96 instrument (Roche Diagnostics) was used to assess relative quantifications of *SERPINA1*. Measured mRNA levels were normalized against a reference gene, cytochrome C1 (*CYC1*), and relative quantifications were determined with the ΔΔ cycle threshold method. Primers and probes were designed with the Universal Probe Library (UPL) Assay Design Center (Roche). Primers and probes for *SERPINA1* were forward 5′-GTGGTTTCTGAGCCAGCAG-3′ and reverse 5′-CCCTGTCCTCGTCCGTATT-3′ (UPL probe #86). Primers and probes for *CYC1* were forward 5′-ATAAAGCGGCACAAGTGGTCA-3′ and reverse 5′-GATGGCTCTTGGGCTTGAGG-3′ (UPL probe #47). Each qRT-PCR measurement was done in triplicate. Statistical analysis was performed by nonparametric Mann–Whitney *U* test with SPSS Statistics 26.0 (IBM Corporation).

### Immunohistochemical staining of AAT

Placental samples were analyzed by immunohistochemical staining to visualize the localization of AAT as described previously [39]. Samples for AAT were from six SPTB deliveries and six STB deliveries. The samples were embedded in paraffin and cut into 4-μm slices. For detection, the samples were incubated with rabbit anti-human AAT antibody (1:1000 dilution, PA5-16661, Invitrogen). Non-immune rabbit IgG was used for negative controls.

### Immuno-EM of AAT and fibronectin

Immuno-EM was performed at the Biocenter Oulu Electron Microscopy Core Facility. Fresh human placenta samples were fixed in 4% paraformaldehyde in 0.1 M phosphate buffer with 2.5% sucrose, pH 7.4 for 6 h. After fixation, samples were immersed in 2.3 M sucrose and frozen in liquid nitrogen. Thin cryosections were cut with a Leica EM UC7 cryo-ultramicrotome (Leica Microsystems). For immunolabeling, sections on Butvar-coated nickel grids were first incubated in 0.1% glycin-PBS for 10 min followed by incubation in a blocking serum containing 1% BSA in PBS for 5 min. We used 1% BSA in PBS for washing steps and dilutions of antibodies and gold conjugates. Sections were exposed to primary antibodies to AAT (1:100 dilution, PA5-16661, Invitrogen) or fibronectin (1:100 dilution, ab6328, Abcam UK) for 45 min. Rabbit anti-mouse IgG (Jackson Immunoresearch Laboratories Inc.) was used as a bridging antibody for fibronectin antibody for 30 min. Bound antibodies were labelled by incubation with protein A–conjugated 10 nm gold (Cell Microscopy Core, University Medical Center Utrecht) for 30 min. Controls were prepared by replacing the primary antibody with PBS. Grids were stained with neutral uranyl acetate (UA) and coated with 2% methyl cellulose containing 0.4% UA. Thin sections were imaged with a Tecnai G2 Spirit 120 kV transmission electron microscope (FEI Company), and images were captured by a Quemesa CCD camera (Olympus Soft Imaging Solutions GMBH).

### Fluorescence colocalization analysis

Florescence colocalization analysis was performed with human placental tissue samples. Human placental tissue samples were treated as described previously [40]. Colocalization of the following complexes were analyzed: AAT–fibronectin, AAT–vitronectin, AAT–collagen, AAT-Rab7, and AAT-CD63.

To detect AAT, fibronectin, vitronectin, collagen IV, Rab7, and CD63, respectively, we used rabbit anti-human AAT antibody (PA5-16661, 1:250 dilution, Thermo Fisher Scientific), mouse anti-human fibronectin antibody (ab6328, 1:250 dilution, Abcam), mouse anti-human vitronectin antibody (MAB88917, 1:250 dilution, Merck), mouse anti-human collagen IV antibody (MA5-13437, 1:250 dilution, Thermo Fisher Scientific), mouse anti-human Rab7 antibody (sc-376362, 1:250, Santa Cruz), and mouse anti-human CD63 antibody (sc-365604, 1:250, Santa Cruz). Primary antibody incubations were done at room temperature for 1 h. Secondary antibodies were goat anti-rabbit IgG Alexa Fluor 488 conjugate (S4412, 1:500 dilution, Cell Signaling Technology) and goat anti-mouse IgG Alexa Fluor 594 conjugate (S8890, 1:500 dilution, Cell Signaling Technology). Tissue samples were observed with a Leica SP8 FALCON laser scanning confocal microscope, and images were acquired with LAS X software. Excitation wavelengths were 499 nm for Alexa Fluor 488 conjugate and 598 nm for Alexa Fluor 594 conjugate. Emission wavelengths were 509–593 nm for Alexa Fluor 488 conjugate and 608–782 nm for Alexa Fluor 594 conjugate. Objectives used were HC PL APO CS2 20×/0.75 DRY and HC PL APO CS2 63×/1.40 OIL. Primary antibodies were omitted in the negative controls.

### Gene knockdown of *SERPINA1* by transfection with siRNA

We used the human placental trophoblast cell line HTR8/SVneo (CRL-3271, ATCC) to perform gene knockdown of *SERPINA1*. Cells were grown in RPMI-1640 growth medium (Thermo Fisher Scientific) supplemented with 10% fetal bovine serum (Sigma-Aldrich) and 1× penicillin/streptomycin (Sigma-Aldrich) under standard culturing conditions (37 °C, 5% CO_2_, humidified atmosphere). Subculturing was done with 0.05% trypsin/0.02% EDTA.

HTR8/SVneo cells were reverse and forward transfected with siRNA targeting *SERPINA1* (s GUCCAUUACUGGAACCUAU, a AUAGGUUCCAGUAAUGGAC). For the control, MISSION siRNA Universal Negative Control #1 (Sigma-Aldrich) was transfected in the same way as siRNA targeting *SERPINA1*. Lipofectamine 3000 reagent (Invitrogen) was used as a transfection reagent. Cells (100,000/well) were incubated with 10 nM siRNA in the reverse transfection. Forward transfection was performed after 24 h of incubation. In the forward transfection, cells were again transfected with 10 nM siRNA concentrations. Cells were incubated with siRNA for 48 h and then harvested with 1× trypsin/EDTA (Sigma-Aldrich).

Knockdown of *SERPINA1* in the silenced cells was verified by qRT-PCR according to the standard qRT-PCR procedure, as described earlier for qRT-PCR of *SERPINA1*. A LightCycler96 (Roche Diagnostics) was used to assess relative quantifications of *SERPINA1*. Statistical analysis was performed by nonparametric Mann–Whitney *U* test with SPSS Statistics 26.0 (IBM Corporation).

### Transcriptomic analysis of *SERPINA1*-silenced HTR8/SVneo cells

Cells were disrupted with a 20-G needle and 1-ml syringe. RNA isolation was done with the RNeasy Micro Kit (Qiagen), and the quality of isolated RNA was checked with an Agilent 2100 Bioanalyzer system at the Biocenter Oulu Sequencing Center, Finland.

RNA sequencing was done at the Finnish Functional Genomics Centre (FFGC; Turku, Finland). Transcriptomes of *SERPINA1*-silenced cells (*n* = 3) and negative control cells (*n* = 3) were determined with the Illumina HiSeq high-throughput sequencing system. Sequencing data were analyzed by the Bioinformatics Unit Core Service at the Turku Centre for Biotechnology, Finland.

### Verification of transcriptomic data from *SERPINA1*-silenced HTR8/SVneo cells by qRT-PCR

Results from the RNA sequencing data of *SERPINA1* knockdown experiments were verified by qRT-PCR in the manner described above. *ACTG1*, *CEACAM1*, *FN1*, and *SLIT2* were chosen from the RNA sequencing data from the *SERPINA1* knockdown. Primers and probes were forward 5′-GCGAGGGATCCTAACTCTCA-3′ and reverse 5′-TGTAGAAGGAGTGGTGCCAGA-3′ for *ACTG1* (UPL probe #9), forward 5′-CCCATCATGCTGAACGTAAA-3′ and reverse 5′-AGGGCCACTACTCCAATCAC-3′ for *CEACAM1* (UPL probe #57), forward 5′-GCGAGAGTGCCCCTACTACA-3′ and reverse 5′-GTTGGTGAATCGCAGGTCA-3′ for *FN1* (UPL probe #52), and forward 5′-CTTCCAGAGACCATCACAGAAA-3′ and reverse 5′-CGTCTAAGCTTTTTATATGGTGAGAA-3′ for *SLIT2* (UPL probe #79). Probes were from the UPL Set (Roche Diagnostics). Each qPCR measurement was done in triplicate. Levels were normalized against the CYC1 level, and relative quantifications were then assessed with the ΔΔ cycle threshold method as described previously.

## Results

### Placental proteomes of preterm birth

**Fig. 2:**
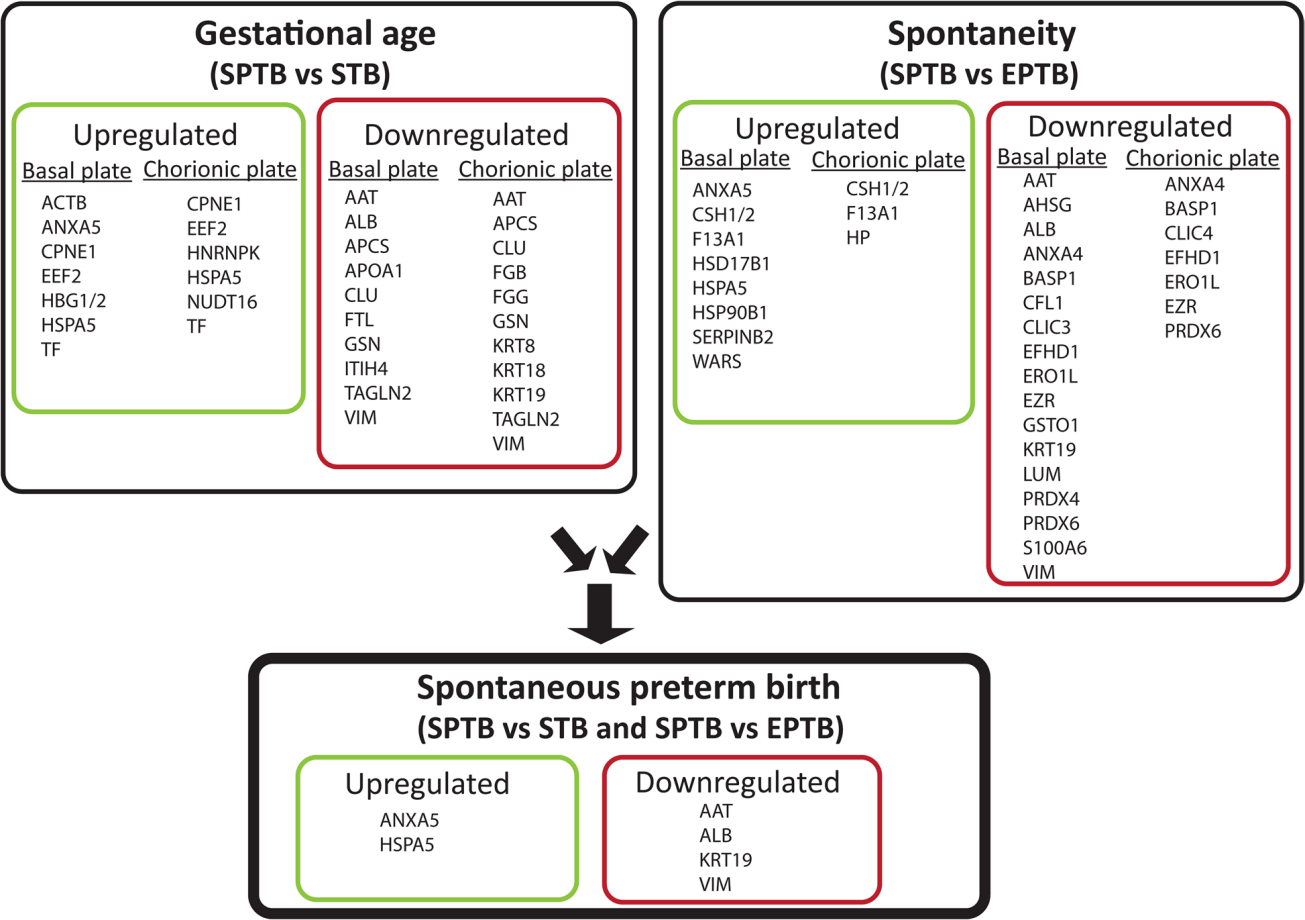
Proteins associated with spontaneous preterm birth. A comparison of spontaneous preterm birth (SPTB) with spontaneous term birth (STB) placental samples identified proteins associated with gestational age. SPTB compared with elective preterm birth (EPTB) placental samples identified proteins associated with spontaneity. Combining comparisons of gestational age and spontaneity identified placental proteins associated with SPTB

**Fig. 3:**
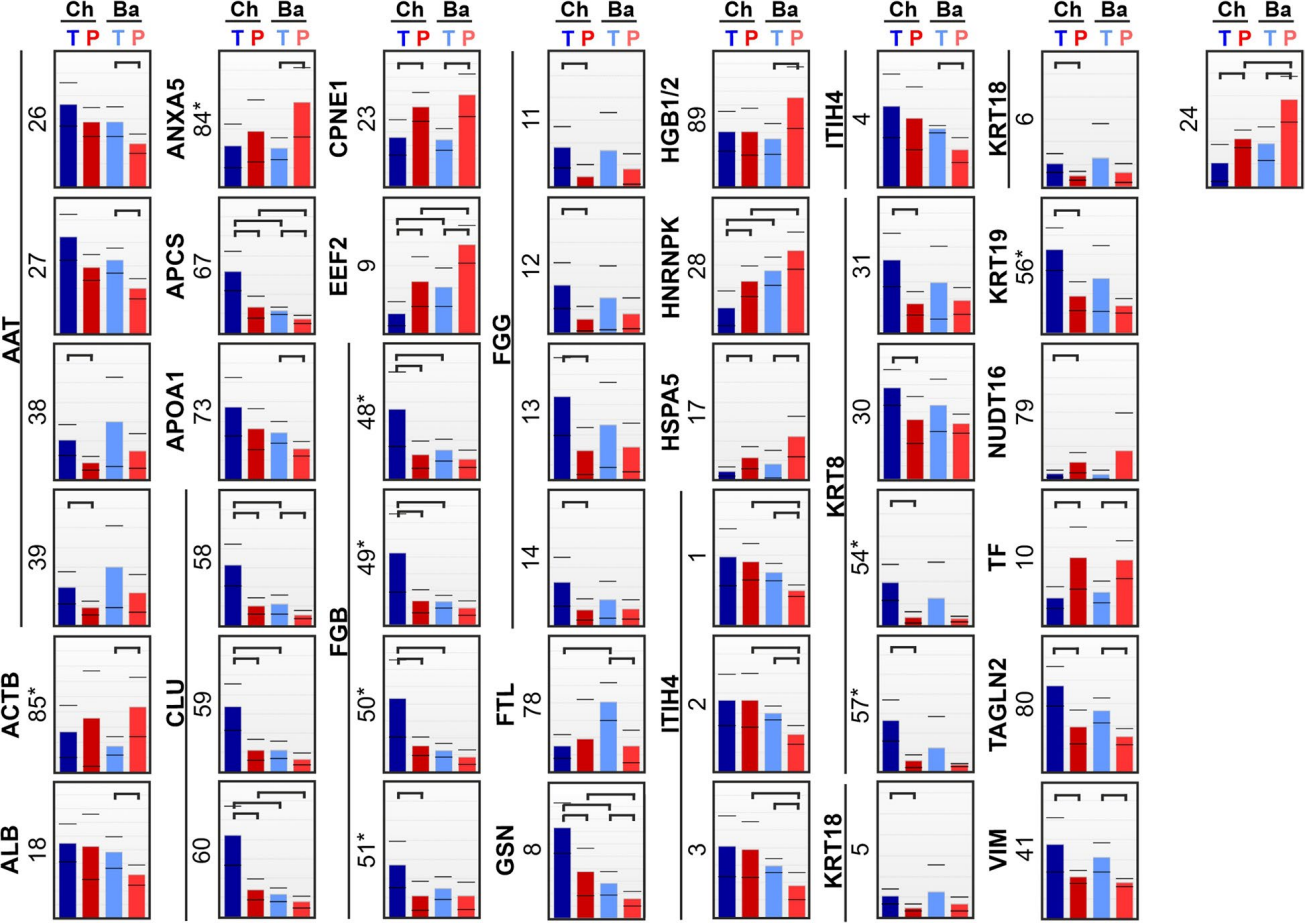
Proteomic differences between spontaneous preterm (P) and term (T) birth in chorionic (Ch) and basal (Ba) plates of the placenta. Exact positions of spots are presented in Additional file: Fig S1. Corresponding differences in protein levels are shown in the expression profile, and statistically significant changes (*p* < 0.05) are indicated by horizontal brackets. Spots corresponding to the same protein are clustered with a vertical line, and protein fragments are indicated with asterisks

Levels of 24 proteins were either up- or downregulated in SPTB vs. STB placentas (Figs. 2 and 3, Table 1). In the basal plate of SPTB placentas, the upregulated proteins were actin cytoplasmic 1 (ACTB), annexin A5 (ANXA5), copine-1 (CPNE1), elongation factor 2 (EEF2), hemoglobin subunit γ-1/2 (HBG1/2), 78-kDa glucose-regulated protein (HSPA5), and serotransferrin (TF). Downregulated proteins in the basal plate of SPTB placentas were alpha-1 antitrypsin (AAT), serum albumin (ALB), serum amyloid P-component (APCS), apolipoprotein A-I (APOA1), clusterin (CLU), ferritin light chain (FTL), gelsolin (GSN), inter-α trypsin inhibitor heavy chain H4 (ITIH4), transgelin-2 (TAGLN2), and vimentin (VIM).

**Table 1:** Differences between spontaneous preterm and spontaneous term birth in the human placental proteome

Spot	Protein	UniProt-KB	Description	Theoretical pI/ MW (kDa)	Detected pI/ MW (kDa)	Ratio	
						T vs P	
						Ch	Ba
**26**	AAT	P01009	α-1-antitrypsin (isoform 1)	5.37 47 (5.37 44)	4.92 60	(− 1.28)	− 1.50
**27**	AAT	P01009	α-1-antitrypsin (isoform 1)		4.97 59	− 1.45	− 1.63
**38**	AAT	P01009	α-1-antitrypsin (isoform 1,2)		4.82 51	− 2.39	(− 2.01)
**39**	AAT	P01009	α-1-antitrypsin (isoform 1,2)		4.86 51	− 2.16	(− 1.82)
85*	ACTB	P60709	Actin, cytoplasmic 1 (fragment)	5.29 42 (5.29 42)	4.93 18	(1.33)	2.48
**18**	ALB	P02768	Serum albumin	5.92 69	5.71 66	(− 1.05)	− 1.52
84*	ANXA5	P08758	Annexin A5 (fragment)	4.93 36 (4.93 36)	4.83 18	(1.35)	2.19
**67**	APCS	P02743	Serum amyloid P-component	6.10 25 (6.12 25)	5.52 25	− 2.42	− 1.62
**73**	APOA1	P02647	Apolipoprotein A-I	5.56 31	5.09 23	(− 1.44)	− 1.52
**58**	CLU	P10909	Clusterin (isoform 3)	6.19 32	4.76 36	− 3.08	− 1.96
**59**	CLU	P10909	Clusterin (isoform 3)		4.82 35	− 2.98	(− 1.75)
**60**	CLU	P10909	Clusterin (isoform 3)		4.88 34	− 2.99	(− 1.49)
**23**	CPNE1	Q99829	Copine-1	5.52 59	5.56 63	1.62	1.95
**9**	EEF2	P13639	Elongation factor 2	6.41 95 (6.42 95)	6.42 95	2.60	1.94
48*	FGB	P02675	Fibrinogen ß chain (C-terminal fragment)	8.54 56 (7.95 51)	5.51 38	− 2.89	(− 1.44)
49*	FGB	P02675	Fibrinogen ß chain (C-terminal fragment)		5.60 39	− 3.04	(− 1.39)
50*	FGB	P02675	Fibrinogen ß chain (C-terminal fragment)		5.71 39	− 2.84	(− 1.44)
51*	FGB	P02675	Fibrinogen ß chain (C-terminal fragment)		5.85 38	− 2.46	(− 1.35)
**11**	FGG	P02679	Fibrinogen γ (isoform γ-B,A)	5.37 52 (5.24 48)	5.41 95	− 3.59	(− 1.99)
**12**	FGG	P02679	Fibrinogen γ (isoform γ-B,A)		5.47 94	− 3.42	(− 1.83)
**13**	FGG	P02679	Fibrinogen γ (isoform γ-B,A)		5.54 94	− 2.91	(− 1.68)
**14**	FGG	P02679	Fibrinogen γ (isoform γ-B,A)		5.58 94	− 2.67	(− 1.57)
**78**	FTL	P02792	Ferritin light chain	5.50 20 (5.50 20)	5.50 20	(1.29)	− 2.73
**8**	GSN	P06396	Gelsolin (isoform 1)	5.90 86 (5.72 83)	5.82 95	− 1.94	− 1.79
**89**	HBG1/2	P69891/2	Hemoglobin subunit gamma-1/2	6.64 16 (6.71 16)	6.36 12	(1.38)	2.55
**28**	HNRNPK	P61978	Heterogeneous nuclear ribonucleoprotein K (isoform 1-3)	5.39 51	5.39 57	2.06	(1.33)
**17**	HSPA5	P11021	78 kDa glucose-regulated protein	5.07 72 (5.01 70)	5.01 71	2.60	2.73
**1**	ITIH4	Q14624	Inter-α-trypsin inhibitor heavy chain H4 (isoform 1)	6.51 103 (6.11 100)	4.90 137	(− 1.08)	− 1.53
**2**	ITIH4	Q14624	Inter-α-trypsin inhibitor heavy chain H4 (isoform 1)		4.92 136	(− 1.00)	− 1.58
**3**	ITIH4	Q14624	Inter-α-trypsin inhibitor heavy chain H4 (isoform 1)		4.94 135	(− 1.05)	− 1.63
**4**	ITIH4	Q14624	Inter-α-trypsin inhibitor heavy chain H4 (isoform 1)		4.96 135	(− 1.18)	− 1.56
**30**	KRT8	P05787	Keratin, type II cytoskeletal 8 (isoform 1,2)	5.52 54	5.24 47	− 1.55	(− 1.34)
**31**	KRT8	P05787	Keratin, type II cytoskeletal 8 (isoform 1,2)		5.33 50	− 2.48	(− 1.54)
54*	KRT8	P05787	Keratin, type II cytoskeletal 8 (isoform 1,2; fragment)		4.85 38	− 5.02	(− 3.82)
57*	KRT8	P05787	Keratin, type II cytoskeletal 8 (isoform 1,2; fragment)		4.79 37	− 4.70	(− 3.49)
**5**	KRT18	P05783	Keratin, type I cytoskeletal 18	5.34 48 (5.34 48)	5.29 102	− 2.03	(− 1.83)
**6**	KRT18	P05783	Keratin, type I cytoskeletal 18		5.35 102	− 2.19	(− 2.07)
56*	KRT19	P08727	Keratin, type I cytoskeletal 19 (C-terminal fragment)	5.05 44	4.74 37	− 2.28	(− 1.99)
**79**	NUDT16	Q96DE0	U8 snoRNA-decapping enzyme (isoform 1)	6.38 21	6.33 21	2.54	(4.78)
**10**	TF	P02787	Serotransferrin	6.81 77 (6.70 75)	6.02 90	2.43	1.95
**80**	TAGLN2	P37802	Transgelin-2 (isoform 1,2)	8.41 22 (8.45 22)	5.59 19	− 1.91	− 1.75
**41**	VIM	P08670	Vimentin	5.05 54 (5.05 54)	4.71 44	− 1.77	− 1.69
**24**	–	–	–	–	5.53 56	2.01	2.04

Spot numbers are according to Additional file: Fig S1. Descriptions and UniProt accession numbers for the identified spots are shown. Spots belonging to the same protein are clustered. Protein fragments are indicated with asterisks. Specific protein isoforms were validated according to spot positions in the gel and the covered protein sequence, as well as isoform-specific peptides obtained by mass spectrometry. Theoretical spot identifications were calculated according to full or mature (in parentheses) protein sequences, whereas detected spot positions in the gel were determined according to selected marker spots. If the identified spot allowed for the presence of several isoforms, theoretical values were indicated for the most common isoform. Ratio shows the change in mean normalized spot volumes between spontaneous preterm (P) and term (T) birth or between basal (Ba) and chorionic (Ch) plates of the placenta. Changes with no significance (*p* > 0.05) are denoted by parentheses. Spot 24 was not identified. For more details about protein levels, statistical significance, and protein identification, see Additional file: Tables S1 and S3. Isoelectric point (pI)

In the chorionic plate of the placentas, CPNE1, EEF2, heterogeneous nuclear ribonucleoprotein K (HNRNPK), HSPA5, U8 snoRNA-decapping enzyme (NUDT16), and TF were upregulated in SPTB; downregulated proteins were AAT, APCS, CLU, fibrinogen β chain (FGB), fibrinogen γ (FGG), GSN, keratin type II cytoskeletal 8 (KRT8), keratin type I cytoskeletal 18 (KRT18), keratin type I cytoskeletal 19 (KRT19), TAGLN2, and VIM.

Ten proteins (AAT, APCS, CLU, CPNE1, EEF2, GSN, HSPA5, TF, TAGLN2, and VIM) out of the identified 24 proteins were up- or downregulated in both sides of the placenta (basal and chorionic plates) (Fig. 2). The rest were differentially regulated in either basal (ACTB, ALB, ANXA5, APOA1, FTL, HBG1/2, and ITIH4) or chorionic (FGB, FGG, HNRNPK, KRT8, KRT18, KRT19, and NUDT16) plates.

### Proteins associated with spontaneous birth in preterm placentas

**Fig. 4:**
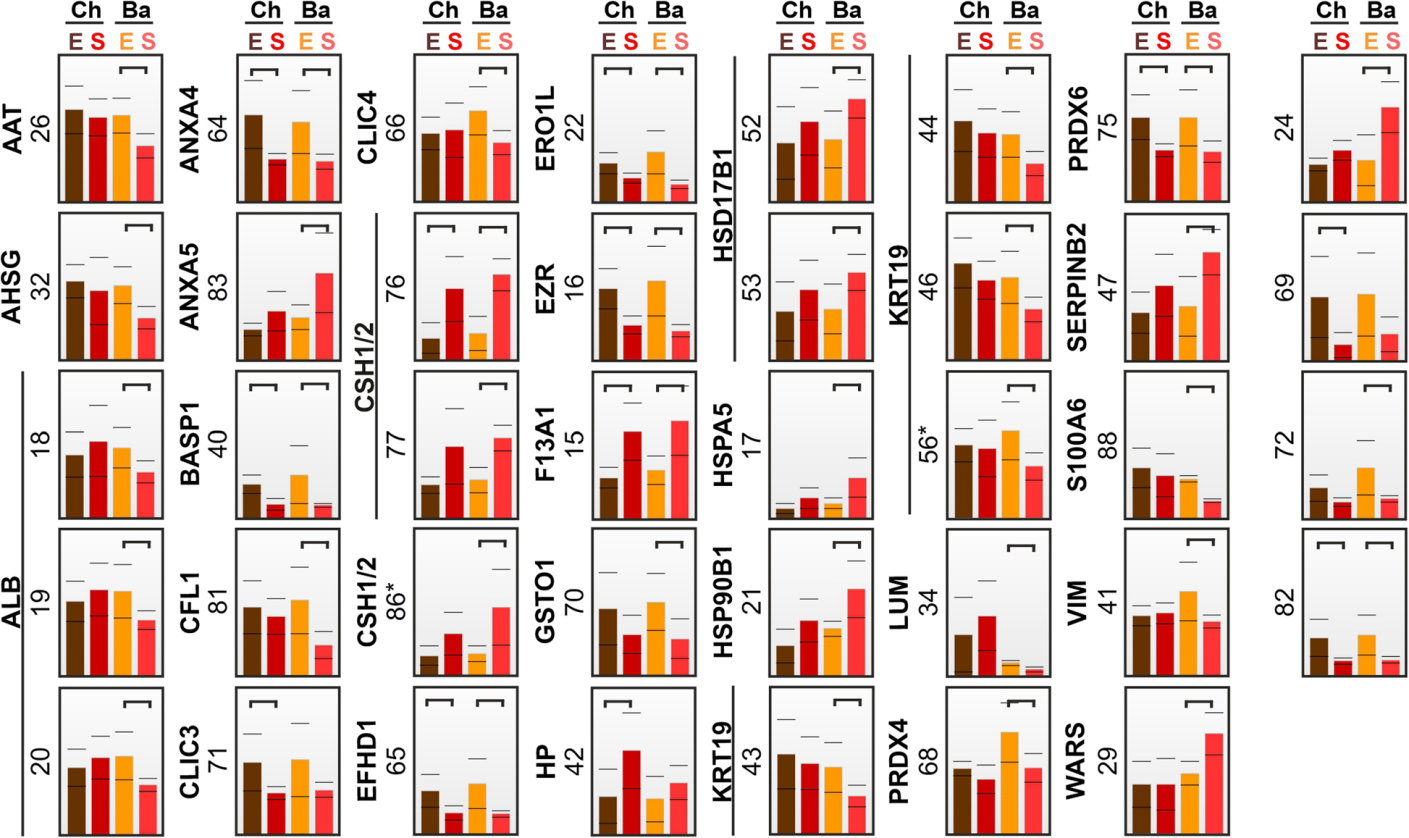
Proteomic differences between spontaneous (S) and elective (E) preterm birth in chorionic (Ch) and basal (Ba) plates of the placenta. Exact positions of spots are presented in Additional file: Fig S1. Corresponding changes in protein levels are shown in the expression profile, and statistically significant changes (*p* < 0.05) are indicated by horizontal brackets. Spots corresponding to the same protein are clustered with a vertical line, and protein fragments are indicated with asterisks

In SPTB and EPTB placental proteomes, 27 proteins either were up- or downregulated (Figs. 2 and 4, Table 2). In the basal plate of SPTB placentas, the upregulated proteins were ANXA5, chorionic somatomammotropin hormone 1/2 (CSH1/2), coagulation factor XIII A chain (F13A1), estradiol 17-β-dehydrogenase 1 (HSD17B1), HSPA5, endoplasmin (HSP90B1), plasminogen activator inhibitor 2 (SERPINB2), and tryptophan-tRNA ligase (WARS). Downregulated proteins in SPTB placental basal plates were AAT, α-2-HS-glycoprotein (AHSG), ALB, annexin A4 (ANXA4), brain acid soluble protein 1 (BASP1), cofilin-1 (CFL1), chloride intracellular channel protein 4 (CLIC4), EF-hand domain-containing protein D1 (EFHD1), ERO1-like protein α (ERO1L), ezrin (EZR), glutathione S-transferase ω-1 (GSTO1), KRT19, lumican (LUM), peroxiredoxin-4 (PRDX4), peroxiredoxin-6 (PRDX6), protein S100-A6 (S100A6), and vimentin (VIM).

**Table 2:** Differences between spontaneous and elective preterm birth in the human placental proteome

Spot	Protein	UniProt-KB	Description	Theoretical pI/ MW (kDa)	Detected pI/ MW (kDa)	Ratio	
						E vs S	
						Ch	Ba
**26**	AAT	P01009	α-1-antitrypsin (isoform 1 or 2)	5.37/ 46.7^1^ (5.37/ 44.3^1^)	4.93 60	(− 1.09)	− 1.53
**32**	AHSG	P02765	α-2-HS-glycoprotein (inactive protein with propeptide)	5.43/ 39.3 (4.53/ 30.2)	4.56 57	(− 1.15)	− 1.77
**18**	ALB	P02768	Serum albumin (isoform 1)	5.92 69 (5.67/ 66.5)	5.71 66	(1.21)	− 1.52
**19**	ALB	P02768	Serum albumin (isoform 1)		5.76 66	(1.15)	− 1.51
**20**	ALB	P02768	Serum albumin (isoform 1)		5.82 66	(1.14)	− 1.57
**64**	ANXA4	P09525	Annexin A4 (isoform 1)	5.83/ 35.9 (5.84/ 35.8)	5.66 30	− 2.03	− 1.97
**83**	ANXA5	P08758	Annexin A5	4.93/ 35.9 (4.93/ 35.8)	4.90 18	(1.58)	2.08
**40**	BASP1	P80723	Brain acid soluble protein 1 (isoform 1)	4.62/ 22.7 (4.62/ 22.6)	4.54 50	− 2.42	− 3.23
**81**	CFL1	P23528	Cofilin-1	8.22/ 18.5 (8.26/ 18.4)	6.01 19	(− 1.15)	− 2.43
**71**	CLIC3	O95833	Chloride intracellular channel protein 3	5.99/ 26.6	5.89 26	− 1.75	(− 1.69)
**66**	CLIC4	Q9Y696	Chloride intracellular channel protein 4	5.45/ 28.8 (5.45/ 28.6)	5.39 26	(1.05)	− 1.56
**76**	CSH1/2	P0DML2/3	Chorionic somatomammotropin hormone 1/2 (isoform 1)	5.35/ 25.0^1^ (5.33/ 22.3^1^)	5.43 22	3.22	3.14
**77**	CSH1/2	P0DML2/3	Chorionic somatomammotropin hormone 1/2 (isoform 1)		5.59 22	(2.17)	2.08
86*	CSH1/2	P0DML2/3	Chorionic somatomammotropin hormone 1/2 (isoform 1, fragment)		5.67 15	(2.07)	3.09
**65**	EFHD1	Q9BUP0	EF-hand domain-containing protein D1 (isoform 1)	5.34/ 26.9	5.22 28	− 2.01	− 2.48
**22**	ERO1L	Q96HE7	ERO1-like protein α	5.48/ 54.4 (5.37/ 51.9)	5.53 63	− 1.66	− 2.78
**16**	EZR	P15311	Ezrin	5.94/ 69.4 (5.95/ 69.3)	5.88 82	− 2.01	− 2.69
**15**	F13A1	P00488	Coagulation factor XIII A chain (inactive protein with propeptide)	5.75/ 83.3 (5.81/ 79.2)	5.74 88	2.09	1.98
**70**	GSTO1	P78417	Glutathione S-transferase ω-1 (isoform 1)	6.24/ 27.6 (6.23/ 27.4)	5.69 27	(− 1.65)	− 2.02
**42**	HP	P00738	Haptoglobin (isoform 1 or 2)		4.79 44	2.19	(1.47)
**52**	HSD17B1	P14061	Estradiol 17-β-dehydrogenase 1	5.46/ 34.9 (5.47/ 34.8)	5.46 35	(1.35)	1.64
**53**	HSD17B1	P14061	Estradiol 17-β-dehydrogenase 1		5.59 35	(1.43)	1.70
**17**	HSPA5	P11021	78 kDa glucose-regulated protein	5.07/ 72.3 (5.01/ 70.5)	5.01 71	(1.99)	2.78
**21**	HSP90B1	P14625	Endoplasmin	4.76/ 92.5 (4.73/ 90.2)	5.09 97	(1.88)	1.85
**43**	KRT19	P08727	Keratin, type I cytoskeletal 19	5.05/ 44.2	4.85 41	(− 1.11)	− 1.74
**44**	KRT19	P08727	Keratin, type I cytoskeletal 19		4.87 41	(− 1.18)	− 1.76
**45**	KRT19	P08727	Keratin, type I cytoskeletal 19		4.90 41	(− 1.22)	(− 1.59)
**46**	KRT19	P08727	Keratin, type I cytoskeletal 19		4.93 41	(− 1.22)	− 1.64
56*	KRT19	P08727	Keratin, type I cytoskeletal 19 (C-terminal fragment)		4.74 37	(− 1.04)	− 1.70
**34**	LUM	P51884	Lumican	6.16/ 38.4 (6.17/ 36.7)	4.51 53	(1.44)	− 1.81
**68**	PRDX4	Q13162	Peroxiredoxin-4	5.86/ 30.5 (5.54/ 26.6)	5.61 25	(− 1.22)	− 1.55
**75**	PRDX6	P30041	Peroxiredoxin-6	6.00/ 25.0 (6.02/ 24.9)	5.82 25	− 1.66	− 1.69
**47**	SERPINB2	P05120	Plasminogen activator inhibitor 2	5.46/ 46.6	5.51 42	(1.55)	1.96
**88**	S100A6	P06703	Protein S100-A6	5.32/ 10.2	4.95 10	(− 1.17)	− 2.19
**41**	VIM	P08670	Vimentin	5.05/ 53.7 (5.05/ 53.5)	4.71 44	(1.05)	− 1.54
**29**	WARS	P23381	Tryptophan-tRNA ligase (isoform 1 or 2)	5.83/ 53.2^1^ (5.83/ 53.0^1^)	5.83 55	(− 1.02)	1.65
**24**	–	–	–	–	5.52 56	1.39	2.39
**69**	–	–	–	–	5.94 29	− 3.92	(− 2.51)
**72**	–	–	–	–	5.15 25	(− 1.79)	− 2.52
**82**	–	–	–	–	4.94 19	− 2.49	− 2.39

Spot numbers are according to Additional file: Fig S1. Descriptions and UniProt accession numbers for the identified spots are shown. Spots belonging to the same protein are clustered. Protein fragments are indicated with asterisks. Specific protein isoforms were validated according to spot positions in the gel and the covered protein sequence, as well as isoform-specific peptides obtained by mass spectrometry. Theoretical spot identifications were calculated according to full or mature (in parentheses) protein sequences, whereas detected spot positions in the gel were determined according to selected marker spots. If the identified spot allowed for the presence of several isoforms, theoretical values were indicated for the most common isoform. Ratio shows the change in mean normalized spot volumes between spontaneous (S) and elective (E) preterm birth in basal (Ba) and chorionic (Ch) plates of the placenta. Changes with no significance (*p* > 0.05) are denoted by parentheses. Spots 24, 69, 72, and 82 were not identified. For more details about protein levels, statistical significance, and protein identification, see Additional file: Tables S2 and S3. Isoelectric point (pI)

In the chorionic plate of SPTB placentas, CSH1/2, F13A1, and haptoglobin (HP) were upregulated; downregulated proteins were ANXA4, BASP1, CLIC3, EFHD1, ERO1L, EZR, and PRDX6. Eight proteins (ANXA4, BASP1, CSH1/2, EFHD1, ERO1L, EZR, F13A1, and PRDX6) out of the identified 27 proteins were up- or downregulated in both the basal and chorionic plates (Fig. 2). The rest were regulated in either the basal (AAT, AHSG, ALB, ANXA5, CFL1, CLIC4, GSTO1, HSD17B1, HSPA5, HSP90B1, KRT19, LUM, PRDX4, SERPINB2, S100A6, VIM, and WARS) or the chorionic (CLIC3 and HP) plate of the placenta.

### Variations in protein expression levels in basal and chorionic plates of SPTB placentas

Comparisons of the two opposing sites of the placenta, the basal and chorionic plates, revealed different protein expression profiles (Additional file: Fig S3, Table 3). There were higher levels of EEF2, HNRNPK, and WARS expression in the basal plate compared to the chorionic plate of SPTB placentas. By contrast, expression levels of AAT, ALB, annexin A3 (ANXA3), APCS, APOA1, complement factor B (CFB), cofillin-1 (CFL1), CLU, GSN, ITIH4, KRT19, lumican (LUM), protein S100-A6 (S100A6), tropomyosin α-1 chain (TPM1), and tropomyosin β chain (TPM2) were lower in the basal plate than in the chorionic plate in SPTB placentas. These findings indicate that there are local variations in protein levels in the placenta. The local variations in AAT, ALB, APOA1, CFB, CFL1, ITIH4, KRT19, and S100A6 levels were observed exclusively in SPTB placentas.

**Table 3:** Proteomic changes between chorionic (Ch) and basal (Ba) plates of human placenta in spontaneous term (T) and preterm (P) birth

Spot	Protein	UniProt-KB	Description	Theoretical pI/MW (kDa)	Detected pI/MW (kDa)	Ratio	
						Ch vs Ba	
						T	P
**Basal plate (Ba) vs chorionic plate (Ch)**							
**25**	AAT	P01009	α-1-antitrypsin (isoform 1)	5.37 47 (5.37 44)	4.85 61	(− 1.32)	− 1.62
**19**	ALB	P02768	Serum albumin (isoform 1)	5.92 69 (5.67 67)	5.76 65	(− 1.16)	− 1.54
**20**	ALB	P02768	Serum albumin (isoform 1)		5.82 66	(− 1.13)	− 1.53
**63**	ANXA3	P12429	Annexin A3	5.62 36 (5.63 36)	5.58 31	− 2.83	− 1.72
**67**	APCS	P02743	Serum amyloid P-component	6.10 25 (6.12 25)	5.52 25	− 2.75	− 1.86
**74**	APOA1	P02647	Apolipoprotein A-I	5.56 31 (5.27 28)	5.18 22	(− 1.67)	− 1.64
**7**	CFB	P00751	Complement factor B (isoform1)	6.67 86 (6.66 83)	5.95 110	(− 1.36)	− 1.86
**81**	CFL1	P23528	Cofilin-1	8.22 19 (8.26 18)	6.01 19	(− 1.32)	− 1.89
**58**	CLU	P10909	Clusterin (isoform 3)	6.19 32	4.76 36	− 2.87	(− 1.84)
**59**	CLU	P10909	Clusterin (isoform 3)		4.82 35	− 2.98	(− 1.75)
**60**	CLU	P10909	Clusterin (isoform 3)		4.88 34	− 3.69	− 1.80
**9**	EEF2	P13639	Elongation factor 2	6.41 95 (6.42 95)	6.42 95	2.32	1.71
48*	FGB	P02675	Fibrinogen ß chain (C-terminal fragment)	8.54 56 (7.95 51)	5.51 38	− 2.45	(− 1.23)
49*	FGB	P02675	Fibrinogen ß chain (C-terminal fragment)		5.60 39	− 3.17	(− 1.46)
50*	FGB	P02675	Fibrinogen ß chain (C-terminal fragment)		5.71 39	− 3.42	(− 1.74)
**78**	FTL	P02792	Ferritin light chain	5.50 20 (5.50 20)	5.50 20	2.75	(− 1.28)
**8**	GSN	P06396	Gelsolin (isoform 1)	5.90 86 (5.72 83)	5.82 95	− 2.59	− 2.39
**28**	HNRNPK	P61978	Heterogeneous nuclear ribonucleoprotein K (isoform 1-3)	5.39 51	5.39 57	2.45	1.64
**1**	ITIH4	Q14624	Inter-α-trypsin inhibitor heavy chain H4 (isoform 1)	6.51 103 (6.11 100)	4.90 137	(− 1.29)	− 1.91
**2**	ITIH4	Q14624	Inter-α-trypsin inhibitor heavy chain H4 (isoform 1)		4.92 136	(− 1.22)	− 2.01
**3**	ITIH4	Q14624	Inter-α-trypsin inhibitor heavy chain H4 (isoform 1)		4.94 135	(− 1.38)	− 2.25
**43**	KRT19	P08727	Keratin, type I cytoskeletal 19	5.05 44	4.85 41	(− 1.48)	− 1.85
**44**	KRT19	P08727	Keratin, type I cytoskeletal 19		4.87 41	(− 1.53)	− 1.79
**45**	KRT19	P08727	Keratin, type I cytoskeletal 19		4.90 41	(− 1.25)	− 1.74
**46**	KRT19	P08727	Keratin, type I cytoskeletal 19		4.93 41	(− 1.35)	− 1.57
**33**	LUM	P51884	Lumican	6.16 38 (6.17 37)	4.45 53	− 5.02	− 9.67
**34**	LUM	P51884	Lumican		4.51 53	− 4.52	− 7.79
**35**	LUM	P51884	Lumican		4.61 53	− 7.59	− 9.41
**36**	LUM	P51884	Lumican		4.71 52	− 5.23	− 5.48
**37**	LUM	P51884	Lumican		4.72 52	(− 2.79)	− 2.96
**88**	S100A6	P06703	Protein S100-A6	5.32 10	4.95 10	(1.12)	− 2.39
**61**	TPM1	P09493	Tropomyosin α-1 chain (isoform 2,3,4)	4.77 27	4.71 33	− 3.03	− 1.99
**62**	TPM1	P09493	Tropomyosin α-1 chain (isoform 1)	4.69 33	4.69 33	− 2.90	− 1.89
**55**	TPM2	P07951	Tropomyosin ß chain (isoform 2)	4.63 33	4.62 35	− 3.91	− 2.75
**29**	WARS	P23381	Tryptophan-tRNA ligase, cytoplasmic (isoform 1)	5.83 53 (5.83 53)	5.83 55	2.29	2.05
**24**	–	–	–	–	5.53 56	(1.80)	1.88
**87**	–	–	–	–	4.74 13	(− 1.10)	− 2.17

Spot numbers are according to Additional file: Fig S1. Spots 24 and 82 were not identified. For more details, see Additional file: Tables S1 and S3

Protein fragments are indicated with asterisks

### AAT/*SERPINA1* associated with preterm birth in proteome and whole exome sequencing data comparisons

We identified six proteins for which levels were associated with SPTB (SPTB vs. STB proteomics with SPTB vs. EPTB proteomics) (Fig. 2): AAT, ALB, ANXA5, HSPA5, KRT19, and VIM. To obtain further evidence of a role for these proteins in spontaneous preterm labor, we searched for rare (minor allele frequency [MAF] < 1%) and common (MAF < 10%) potentially damaging variants (categories 1–3) in *ALB*, *ANXA5*, *HSPA5*, *KRT19*, *SERPINA1*, and *VIM* in whole exome sequencing (WES) data. The WES data included maternal exomes from Northern Finnish and Danish population sets and from the fetal exomes of the Northern Finnish population set. Of *ALB*, *ANXA5*, *HSPA5*, *KRT19*, *SERPINA1*, and *VIM*, only the exonic variants of *HSPA5* and *SERPINA1* were found in at least two different families (Table 4). In total, there were three variants of *SERPINA1* in four different families and one variant of *HSPA5* in two families. All of these variants were predicted to be damaging by the SIFT and PolyPhen-2 tools (Table 4). As the role of HSPs in SPTB was studied previously [35], we focused on AAT/*SERPINA1*.

**Table 4:** Variants of *HSPA5* and *SERPINA1* identified by whole exome sequencing

Gene symbol	Country	Family ID	Status	Chr^**a**^	Reference allele	Sample allele	rsID	Protein variant	MAF^**b**^ European Finnish	MAF^**b**^ European non-Finnish	SIFT Function Prediction	PolyPhen-2 Function Prediction
*HSPA5*	Finland	24	Mother	9	T	C	rs56136100	E557G	0.01341	0.00213	Damaging	Benign
*HSPA5*	Denmark	60	Mother	9	T	C	rs56136100	E557G	0.01341	0.00213	Damaging	Benign
*SERPINA1*	Finland	159	Mother	14	C	T	rs28929474	E366K	0.01796	0.01835	Damaging	Probably damaging
*SERPINA1*	Finland	159	Child	14	C	T	rs28929474	E366K	0.01796	0.01835	Damaging	Probably damaging
*SERPINA1*	Finland	159	Child	14	C	T	rs28929474	E366K	0.01796	0.01835	Damaging	Probably damaging
*SERPINA1*	Finland	210	Child	14	C	T	rs28929474	E366K	0.01796	0.01835	Damaging	Probably damaging
*SERPINA1*	Finland	185	Mother	14	C	T	rs121912712	E387K	0.00278	0.00007	Damaging	Benign
*SERPINA1*	Denmark	25	Mother	14	G	A	rs28929470	R247C	0.00056	0.00418	Damaging	Possibly damaging

^a^Chromosome

^b^Minor allele frequency

### Rare, potentially damaging *SERPINA1* variants in families with recurrent preterm birth

**Fig. 5:**
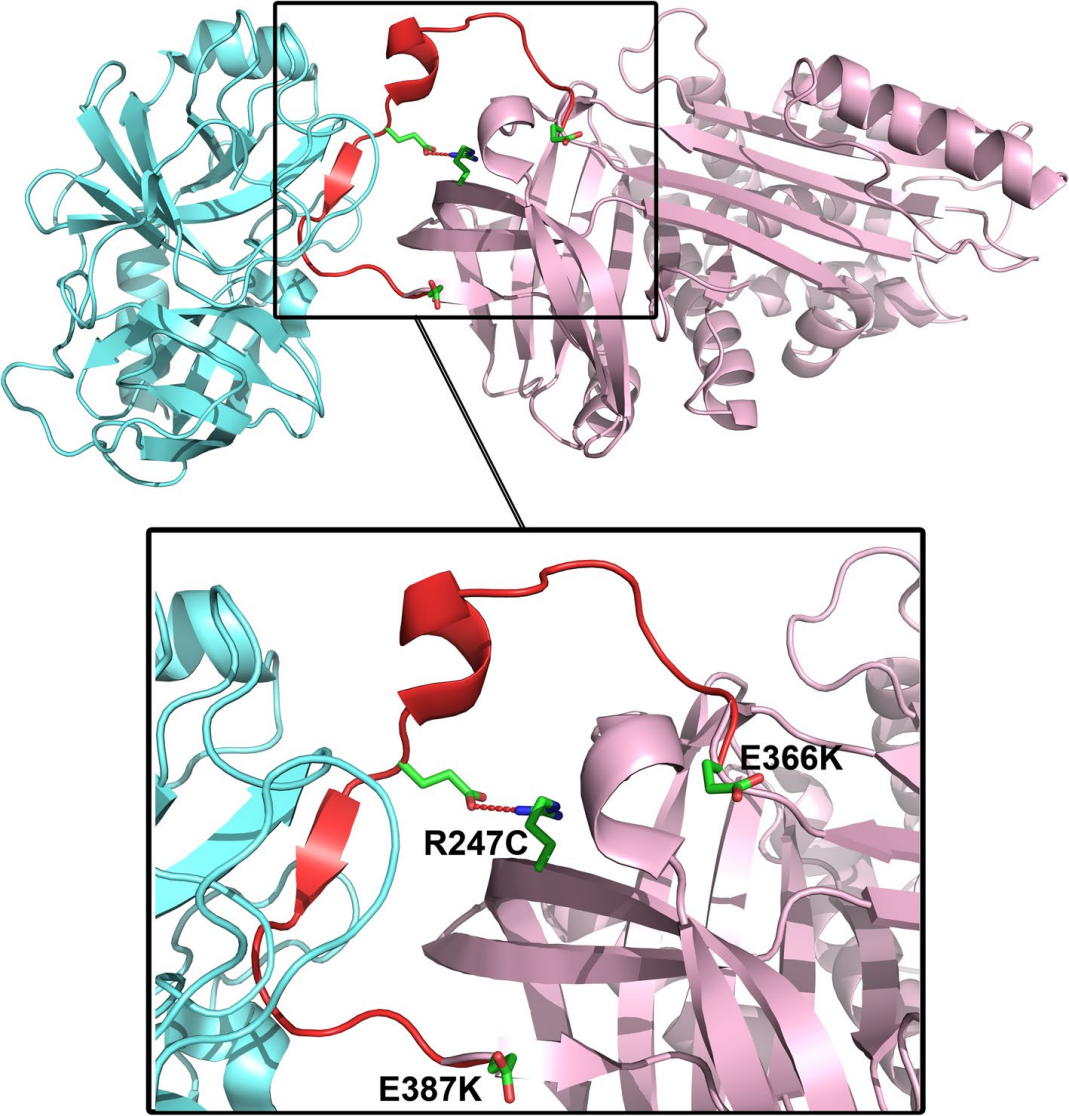
Crystal structure of human AAT bound to cattle trypsin (PDB code 1OPH, [46]). Structure is drawn as ribbons, and AAT and trypsin are colored in pink and cyan, respectively. The region of AAT that interacts with trypsin is highlighted in red; this region is known as the reactive center loop [45]. Variants of *SERPINA1* identified via whole exome sequencing of preterm birth cases (this study) cause amino acid changes Arg247Cys (R247C), Glu366Lys (E366K, Z variant), and Glu387Lys (E387K, F variant) in AAT. Figure prepared with PyMOL

Two out of three *SERPINA1* variants from the WES data were considered likely to affect the function of AAT (Fig. 5). Variant rs28929474 (E366K, also known as Z variant) changes a glutamate into lysine, which results in unstable AAT and retention in the endoplasmic reticulum with a reduction in serum levels [41–43]. Variant rs28929470 (R247C, also known as F variant) has no effect on AAT serum levels but does result in a reduced association with the target protease [44, 45] because of loss of an important electrostatic interaction when arginine is substituted for cysteine (Fig. 5). Thus, the *SERPINA1* variants identified by WES affect the functionality of AAT. Moreover, *SERPINA1* has approximately 200 significant expression quantitative trait loci (eQTLs) in GTEx v8 data (e.g., in whole blood, cultured fibroblasts, and across tissues) that may contribute to the lower expression levels of *SERPINA1* observed in the SPTB cases included in the proteomics data. These findings suggest that either low expression levels of *SERPINA1* or dysfunctional AAT may be predisposing factors for premature birth.

### Low protein and mRNA levels of AAT/*SERPINA1* are associated with SPTB

To investigate the role of AAT in the placenta, we carried out a western blot analysis of AAT expression in samples taken from different sites of the placenta (SPTB *n* = 6 placentas, STB *n* = 6 placentas) (Additional file: Fig S2). As in placental proteomics, western blot analysis revealed that AAT was downregulated in SPTB compared to STB placentas, particularly in the chorionic plate (*p* = 0.006). In SPTB vs. STB proteomics, AAT1 expression levels were significantly different: 1.6-fold (*p* = 0.002) and 2.4-fold (*p* = 0.01) in the basal and chorionic plates, respectively.

**Fig. 6:**
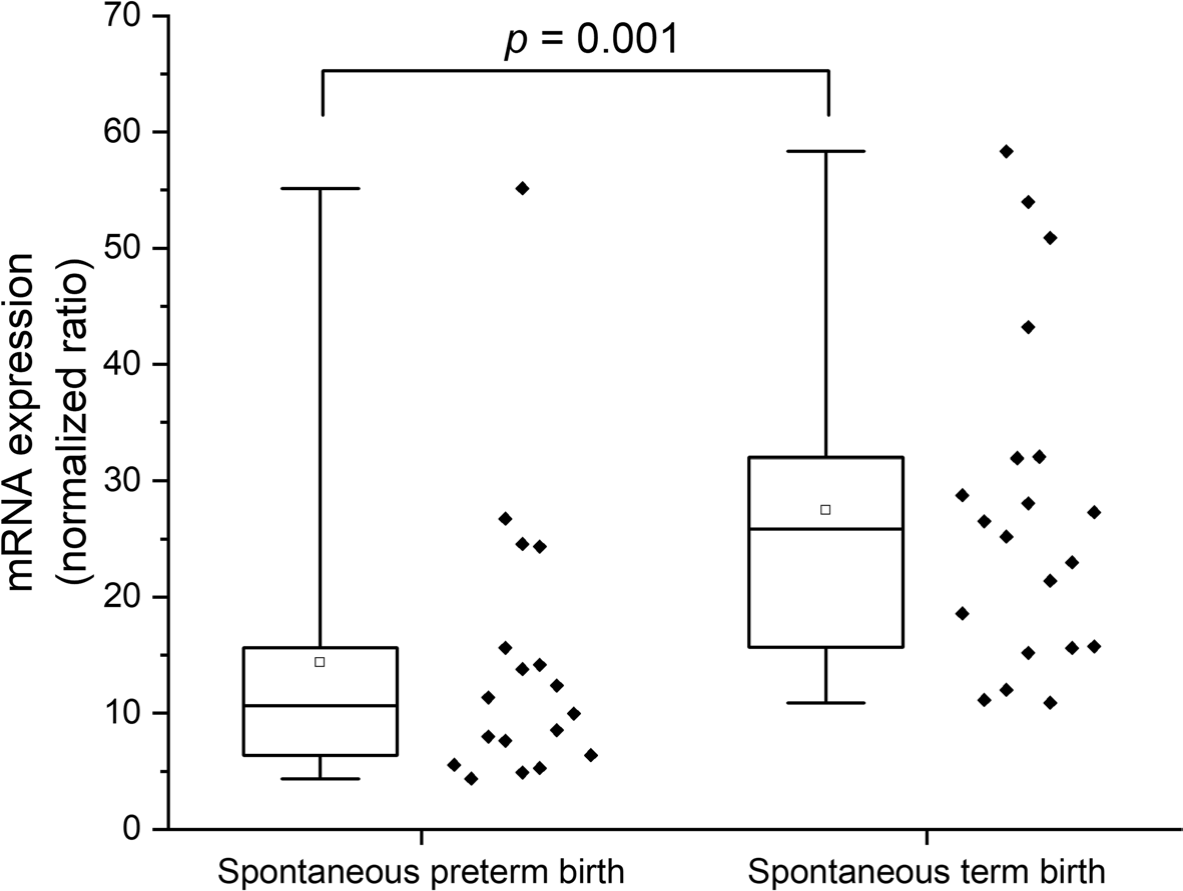
Placental mRNA expression of *SERPINA1* in spontaneous preterm birth and spontaneous term birth. Relative mRNA expression of *SERPINA1* in basal plate of the placenta. mRNA levels were normalized against the housekeeping gene *CYC1*. Statistical analysis was performed with Mann–Whitney *U* test to discover differences. Quartiles represented by box and whiskers. Ends of whiskers represent minimum and maximum values. Inside the box, median is indicated with a line and mean value is represented with a square

Next, we studied whether AAT is regulated at the transcriptional level by analyzing mRNA expression of *SERPINA1* in placental samples from SPTB (*n* = 18) and STB (*n* = 20) by qRT-PCR. *SERPINA1* mRNA expression was lower in SPTB compared to STB placentas (*p* = 0.001, fold change [FC] = −1.91; Fig. 6). qRT-PCR of *SERPINA1* indicated that low levels of both AAT/*SERPINA1* protein and mRNA were associated with SPTB.

### AAT colocalizes with fibrinoid deposit components in placenta

**Fig. 7:**
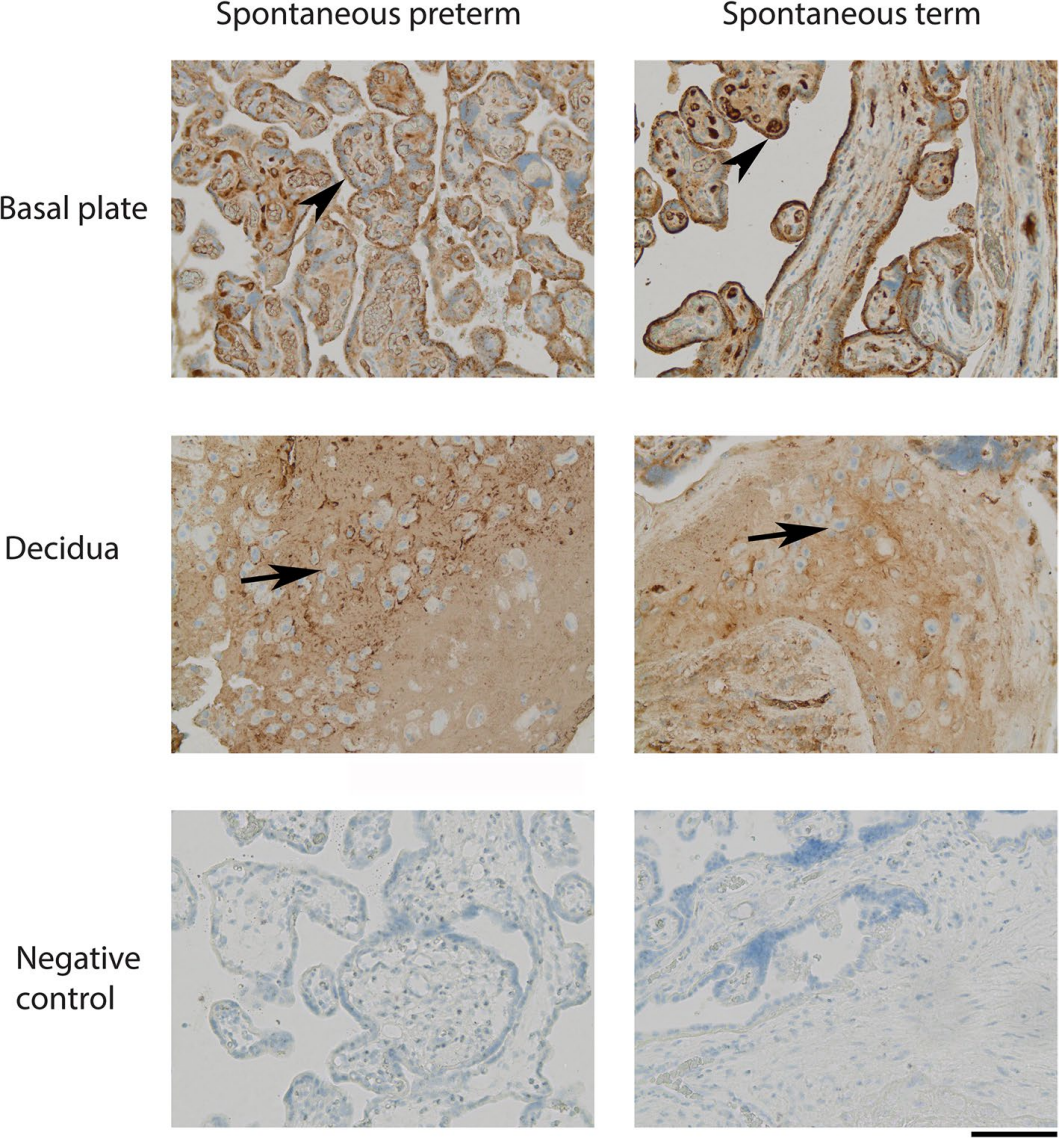
Placental localization of AAT in spontaneous preterm (SPTB) and term (STB) births. Samples from SPTB and STB placentas were immunostained with anti-human AAT antibody. Samples were from the basal plate (maternal side) and from the decidua of the placenta. Immunostaining is indicated by arrowheads in villous trophoblasts (cytotrophoblasts and syncytiotrophoblasts) and arrows in decidual trophoblasts. Original magnification × 20 in all figures. Control is isotype control for immunostaining. Scale bar: 100 μm

**Fig. 8:**
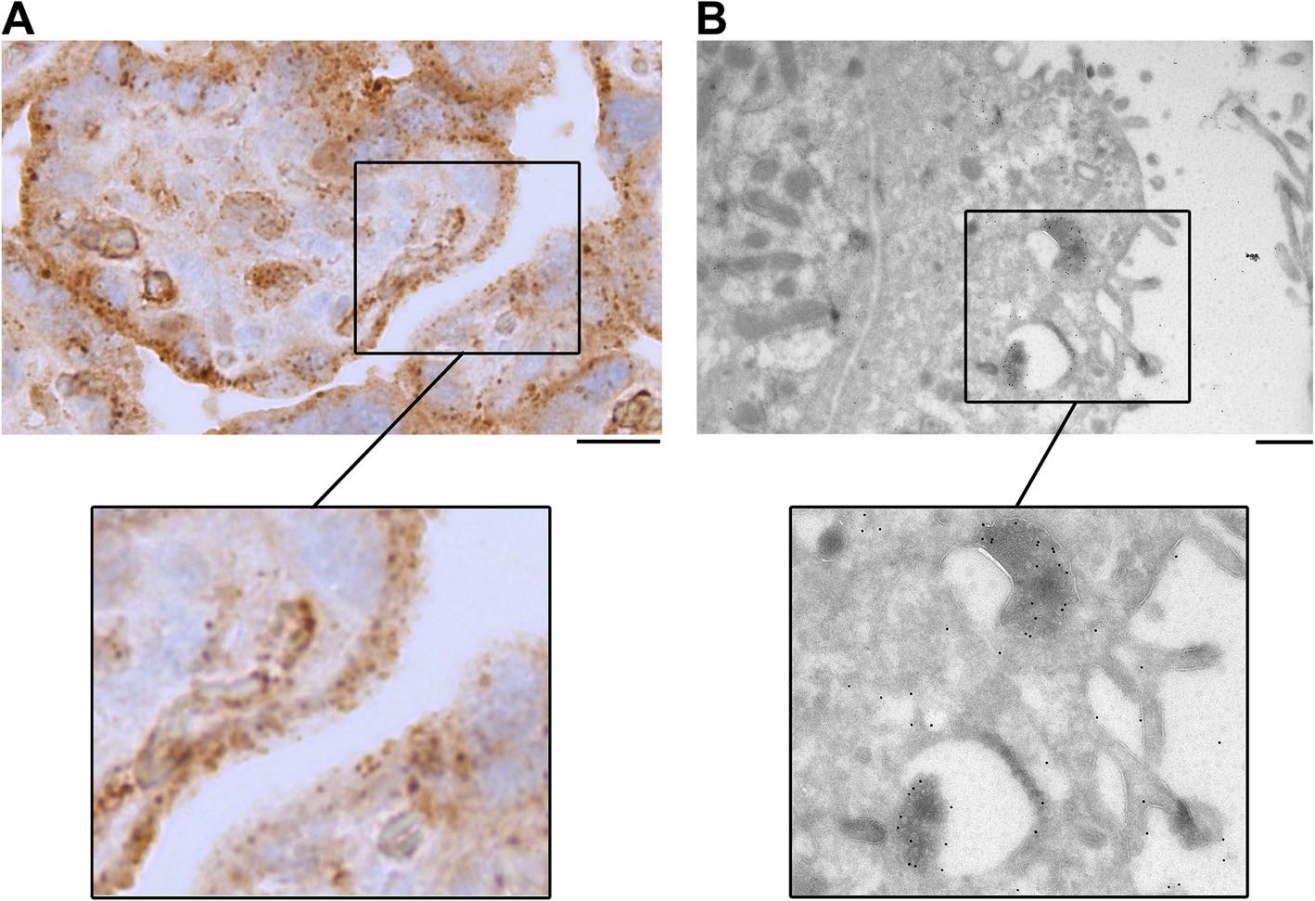
Stained cytoplasmic AAT granules in syncytiotrophoblasts of spontaneous term birth placenta. In both **A** and **B**, samples from the basal plate (maternal side) of the placenta immunostained with anti-human AAT antibody. **A** Immunohistochemistry image of AAT staining in syncytiotrophoblasts. Scale bar: 20 μm. **B** Immunoelectron microscopy image of AAT staining in syncytiotrophoblasts. Scale bar: 0.5 μm

We used immunohistochemistry to assess the localization of AAT in placental tissues. Samples of the basal plate from SPTB and STB deliveries were stained with anti-human AAT antibody (Fig. 7). Staining was strong for AAT in cytotrophoblasts and syncytiotrophoblasts in the basal plate. We also observed AAT in the extracellular matrix of the decidua and AAT-stained granules in the cytosol of cyto- and syncytiotrophoblasts (Figs. 7 and 8A).

Next, we used immunoelectron microscopy (immuno-EM) to visualize more precisely the intracellular localization of AAT in placental trophoblasts and in the decidua. The AAT granules seen in cyto- and syncytiotrophoblasts (Fig. 8A) originated from densely packed AAT inside vesicles (Fig. 8B). The dense bodies, vesicles, and surface microvilli suggest that these syncytiotrophoblasts are involved in endocrine activity [47, 48]. In the extracellular matrix, we observed AAT in fibrillar networks (Additional file: Fig S4A and C) and in dense structures (Additional file: Fig S4B) that may be decidual fibrinoid deposits. These observations suggest that the protease inhibitor activity of AAT may play a role in regulating the amount and composition of fibrinoid deposits in the human placenta.

To further characterize the AAT-containing granules, we did fluorescence colocalization analysis with AAT- and exosome/endosome-specific antibodies. We used Rab7 and CD63 as endosome and exosome markers, respectively. Rab7 is known particularly as a late endosome and lysosome marker. As seen in Additional file: Fig S5, AAT and Rab7 did not colocalize. Thus, the granules containing AAT were not late endosomes or lysosomes. By contrast, AAT and CD63 colocalized in the same areas in the villi, which suggests that the granules were exosomes. However, 3D analysis of the images suggested that AAT and CD63 were not within the same granules (Additional file: Fig S5). These results did not allow us to determine the type of granules in which AAT was located.

Decidual fibrinoid deposit, also known as matrix-type fibrinoid, is secreted by decidual trophoblasts and consists of collagen, laminin, vitronectin, and fibronectin including fetal fibronectin [49]. In addition to anti-AAT staining, we performed immuno-EM staining with an anti-human fibronectin antibody that does not recognize plasma fibronectin. Immuno-EM staining revealed that fibronectin was mostly found in fibrillary networks (Additional file: Fig S4A and C), which suggests that these placental regions are fibrinoid deposits. These results also indicate that AAT is found in decidual fibrinoid deposits.

Next, we further characterized the fibrinoid structures in which AAT was localized by fluorescence colocalization analysis. There are two main subtypes of fibrinoids, fibrin type and matrix type, which differ in structure, composition, and function [50]. Fibrin-type fibrinoid is the result of blood clotting and is mostly composed of fibrin, albumin, immunoglobulins, and different types of fibronectins. Matrix-type fibrinoid is a secretory product of decidual trophoblast cells and is composed of collagen type IV, laminin, heparan sulfate, vitronectin, and fibronectins [49, 50]. EM analysis revealed that AAT may be associated with matrix-type fibrinoids.

**Fig. 9:**
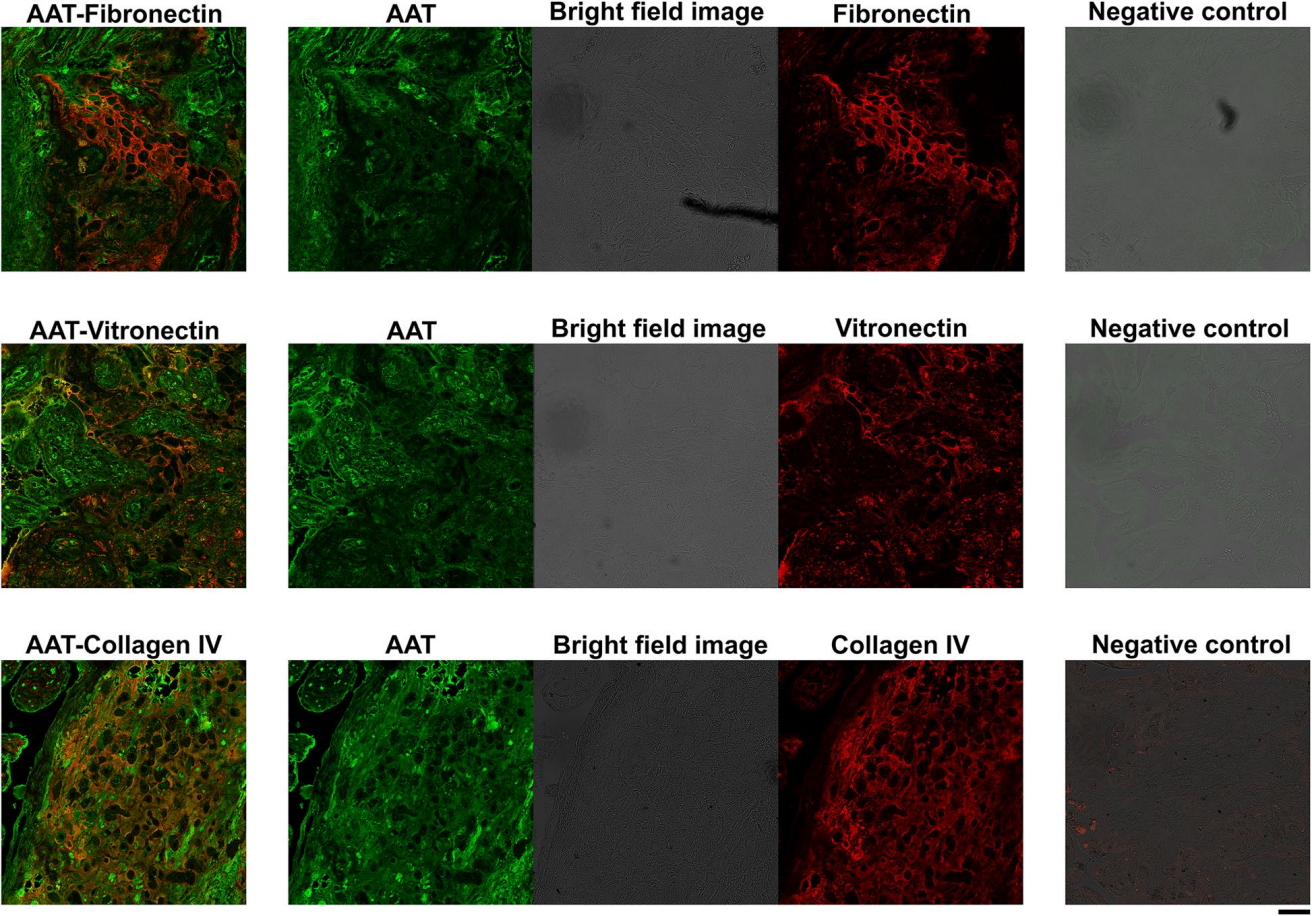
Confocal microscopy images of human placenta expressing endogenous AAT, fibronectin, vitronectin, and collagen IV. Colocalization fluorescence image of AAT (green) and fibronectin (red) (top row), AAT (green) and vitronectin (red) (middle row), and AAT (green) and collagen IV (red) (bottom row). Middle columns are separate green channel, red channel, and brightfield images, which are shown merged in left column. Panels in right column show negative controls treated the same way as samples but with primary antibody omitted. Objective lens: HC PL APO CS2 × 20/0.75 DRY. Scale bar: 10 μm

There are three types of compartments in matrix-type fibrinoid [49]. Subtype 1 is densely granular and basal lamina-like and composed of collagen IV and laminin [49, 50]. To determine if AAT is located in matrix-type fibrinoid subtype 1 deposits, we stained tissue with both AAT- and collagen IV-specific antibodies. As seen in Fig. 9, localization of AAT and collagen IV was similar and mostly around decidual trophoblasts, whereas there was only a modest overlay with the subtype 2 marker vitronectin and the subtype 3 marker fibronectin. Thus, fluorescence colocalization analysis revealed that AAT colocalizes predominantly with subtype 1 fibrinoid deposits in placenta.

### siRNA-induced knockdown of *SERPINA1* indicates that *SERPINA1* regulates actin cytoskeleton pathway and extracellular matrix organization

To investigate potential functions of *SERPINA1* in placental cells, we silenced *SERPINA1* expression in the HTR-8/SVneo human trophoblast cell line with small interfering RNAs (siRNAs). Based on qRT-PCR, silencing efficacy was 94% (Additional file: Fig S6). Next, we characterized the transcriptomes of HTR-8/SVneo cells in which *SERPINA1* was silenced, as well as of cells treated with siRNA Universal Negative Control #1. The threshold we used for the analysis was a false discovery rate (FDR)–adjusted *p* value of ≤0.05 and an FC of ≥1.5. Transcriptomic data analysis identified 442 upregulated and 623 downregulated genes after *SERPINA1* knockdown compared to samples treated with siRNA Universal Negative Control. Up- and downregulated genes that had fold changes of >2.0 are in Additional file: Tables S4 and S5.

KEGG pathway database analyses (Additional file: Table S6) identified regulation of actin cytoskeleton as the most affected pathway after *SERPINA1* knockdown (*p* = 0.00013). Altogether, 26 genes related to regulation of actin cytoskeleton were affected by *SERPINA1* knockdown. Among the affected genes was fibronectin 1(*FN1*). fFN is one of the best predictors of preterm birth in all populations studied to date [51].

A Gene Ontology (GO) Biological Processes search (Additional file: Table S7) identified positive regulation of cell migration (27 genes) and extracellular matrix organization (27 genes) as processes that are affected by *SERPINA1* silencing. A GO Cellular Component (GO-CC) search showed that *SERPINA1* knockdown particularly affected genes that encode proteins found in the proteinaceous extracellular matrix (32 genes) and extracellular exosomes (186 genes) (Additional file: Table S8).

The gene most strongly regulated by *SERPINA1* knockdown was actin gamma 2, smooth muscle (*ACTG2*). ACTG2 is involved in processes such as Slit–Robo signaling in development and actin nucleation by the ARP–WASP complex. A paralog of *ACTG2*, actin alpha cardiac muscle 1 (*ACTC1*), was also among the upregulated genes (*p* = 0.0001, FC = 3.43). Like ACTG2, ACTC1 is involved in Slit–Robo signaling in development. Moreover, *SLIT2* (*p* = 0.0009, FC = 2.08) and *SLIT3* (*p* = 0.0006, FC = 2.28) were among the upregulated genes after *SERPINA1* silencing. We previously showed that Slit–Robo signaling has a role in SPTB (Tiensuu et al. 2019); thus, we investigated possible interactions further.

qPCR showed that *SLIT2* is upregulated (*p* = 0.004) [39] and *SERPINA1* downregulated (Additional file: Fig S7) in SPTB placentas. Based on these findings, we asked whether mRNA levels of *SERPINA1* and *SLIT2* correlated in the placenta. When we compared qPCR results using exactly the same placenta specimens, we found that *SERPINA1* and *SLIT2* levels were significantly correlated (*p* = 0.006). These data indicate that S*ERPINA1* may be an important regulator of *SLIT2* and, thus, Slit–Robo signaling.

To verify our findings from the RNA sequencing data, we used qRT-PCR to analyze the effect of *SERPINA1* knockdown on expression levels of selected genes from a larger number of specimens (*n* = 6). We verified the results for *ACTG2*, one of the downregulated genes (carcinoembryonic antigen-related cell adhesion molecule 1 [*CEACAM1*]), one gene from the regulation of actin cytoskeleton pathway (*FN1*), and *SLIT2*. According to the sequencing data, *FN1* and *SLIT2* were both upregulated after *SERPINA1* knockdown.

The qRT-PCR results were in line with those from transcriptome sequencing (Additional file: Table S9). Knockdown of *SERPINA1* downregulated mRNA expression of *CEACAM1* (Additional file: Fig S7) and upregulated *ACTG2*, *FN1*, and *SLIT2* mRNA expression (Additional file: Fig S7), similar to the results of RNA sequencing. Thus, the RNA sequencing and qRT-PCR results are in accordance and confirm that *SERPINA1* knockdown affects the expression genes associated with Slit–Robo signaling and regulation of the actin cytoskeleton in a cell culture model.

## Discussion

Evaluation of the risk of giving birth preterm in human pregnancy is challenging due to a lack of accurate biomarkers and an incomplete knowledge of the mechanisms involved in the onset of SPTB [6]. According to epidemiological evidence, both maternal and fetal genomes are involved in regulation of the length of pregnancy [52]. The placenta and fetal membranes contain cells of both maternal and fetal origin that are in direct contact with each other [50], which requires immune tolerance to foreign antigens. Switch from anti-inflammatory state to proinflammatory state occurs in the normal delivery. Spontaneous preterm birth is regarded as a syndrome resulting from multiple causes, including infection or inflammation. Thus, it is likely that SPTB is the result of the changes in the infection or inflammation-related factors and pathways. The potential role of the placenta in regulation of the length of pregnancy was a focus of the present study.

Here, we analyzed the proteomes of the basal and chorionic plates of SPTB, STB, and EPTB placentas to identify proteins associated with both spontaneity and gestational age. We then identified proteins (ALB, ANXA5, HSPA5, KRT19, AAT, and VIM) associated with SPTB. The entire exons of these six proteins were then analyzed for potentially damaging variants in families with recurrent preterm births. Of the six candidates revealed by proteomics, *SERPINA1* and *HSPA5* had at least two potentially damaging variants. One *HSPA5* variant was found in two families and two damaging *SERPINA1* variants were found in three families. The *SERPINA1* locus is highly polymorphic [43, 53]; a genome-wide association study found several *SERPINA1/SERPINA6* locus variants that affect plasma cortisol levels, including the T allele at rs12589136, which is associated with higher plasma cortisol levels [54]. These findings suggest a role for AAT/*SERPINA1* in the initiation of labor and pregnancy complications and led us to study AAT/*SERPINA1* in more detail.

The three WES variants that we identified in Finnish and Danish SPTB cases affect the function of AAT. Variant rs28929474 (Z variant) changes glutamate into lysine (E366K) [55], while variant rs28929470 (F variant) changes arginine into cysteine (R247C) [43, 55]. A third variant of *SERPINA1*, rs121912712, changes glutamate into lysine (E387K) and has not yet been reported to be associated with disease. All three variants, however, are located in a loop known as the reactive center loop (RCL) [45]. The RCL is the region of AAT that binds to the target protein, which results in the inhibition of proteases. The Z variant has the most effect on the stability of AAT. This variant resides in the endoplasmic reticulum [41–43] and is eventually degraded, which results in reduced anti-protease activity. The F variant does not influence the amount of AAT; instead, it reduces the binding affinity of AAT to proteases. With both Z and F variants, protease is not properly inhibited, which allows protease activity to continue [56].

The unstable Z variant of AAT has been reported to escape intracellular pathways of protein degradation by forming AAT granules [43]. In immuno-EM images, we observed AAT granules inside of cyto- and syncytiotrophoblasts in both SPTB and STB placentas. This suggests that similar granules are part of the common secretory pathway of AAT. Based on our immunohistology and immuno-EM analyses, we propose that AAT is moving out of these cells into the intervillous space, which consists of maternal blood. Moreover, in the analysis of *SERPINA1* silencing affected genes, extracellular exosome was the most enriched GO term. These are in line with the observation that AAT levels increase in the blood of the mother during pregnancy [57]. Overall, these dense bodies and vesicles are evident in syncytiotrophoblasts and they are the site of metabolic activity [47, 48].

In addition to its production by villous trophoblasts, AAT is produced by the liver and the lung. Types of cells that produce AAT include macrophages and neutrophils. AAT levels in maternal blood are exceptionally low in spontaneous abortions [21]. However, it is unknown which tissues or cells contribute the most to elevated AAT levels in maternal blood during pregnancy. A study of blood monocytes revealed that the degree of *SERPINA1* promoter methylation decreases progressively during pregnancy [58]. Furthermore, AAT serum levels are several-fold higher in the end of pregnancy than during the first trimester [57]. Our placental proteomic findings show that low AAT content in the placenta is associated with SPTB. According to our findings, *SERPINA1* mRNA levels are associated with those of AAT protein levels in the placenta, regardless of the duration of pregnancy or labor. This suggests that AAT is regulated at the mRNA level, which leads to decreased levels of the protein in SPTB. The lower AAT/*SERPINA1* levels in SPTB may be due to exonic variants or eQTL SNPs that affect expression. Additionally, certain microRNAs could regulate *SERPINA1* mRNA levels.

To understand more about the function of AAT in trophoblast cells, we silenced its expression in a continuous human cell line of extravillous invading trophoblasts (HTR8/SVneo). Altogether, 1065 genes were affected at least 1.5-fold by *SERPINA1* siRNA knockdown. Our silencing experiment indicates that *SERPINA1* regulates many genes of the Slit–Robo signaling pathway. We previously showed that Slit–Robo signaling has a potential role in SPTB [39]; in this genome-wide study, a *SLIT2* variant was associated with SPTB, and in trophoblasts, Slit–Robo signaling affected the expression of immune response–modifying genes like inflammatory cytokines and chemokines, which have proposed roles in the onset and progression of spontaneous preterm labor (5). Additionally, AAT was previously shown to induce anti-inflammatory cytokines [21, 58, 59]. By contrast, our *SERPINA1* silencing experiment did not indicate that cytokines were extensively affected by *SERPINA1* silencing. However, AAT*/SERPINA1* may regulate genes related to infection, inflammation, and immune response through regulation of the Slit–Robo signaling pathway.

Pathway analysis of genes affected by *SERPINA1* silencing identified regulation of actin cytoskeleton as the top pathway: 26 genes in this pathway were affected by *SERPINA1* silencing. Human placental syncytiotrophoblast microvilli are supported by an underlying cytoskeleton that consists mainly of actin microfilaments [60]. The microvilli contain a core of actin filaments running from the tip of the microvillus to the apical cytoplasm. The surface of the syncytial trophoblast is covered by a microvillous (brush) border that is in direct contact with maternal blood. Thus, it is the site where a variety of transport, enzymatic, and receptor activities take place. Organization of the brush border membrane, as well as other features of placental villus organization, could be influenced by the distribution of cytoplasmic actin filaments [61]. Therefore, by affecting the acting cytoskeleton, AAT may influence placental villus organization and its diverse functions.

A GO search identified the top GO terms affected by *SERPINA1* knockdown as positive regulation of cell migration and extracellular matrix organization and revealed that *SERPINA1* knockdown particularly affected genes that encode proteins secreted into extracellular spaces. Among these genes were collagen alpha-1 (IV) chain (*COL4A1*), laminin subunit alpha 2 (*LAMA2*), laminin subunit alpha-4 (*LAMA4*), and *FN1*. Proteins encoded by these genes are present in different types of fibrinoid deposits in the placenta [49]. This suggests that AAT has a role in extravillous fibrinoid matrix organization. The presence of AAT in the placenta was demonstrated previously in preeclampsia cases, in which AAT is particularly prominent in the fibrinoid region of the decidua and in villous trophoblasts [62]. In placentas from SPTB pregnancies, in addition to its presence in cyto- and syncytiotrophoblasts, we also observed AAT in the decidual fibrinoid matrix. In preeclampsia cases, increased AAT levels in the decidua are associated with more fibrinoid deposits in the same region [62]. Similarly, we propose that lower levels of AAT result in lower levels of fibrinoid deposits, as AAT levels were lower in biopsies from the basal plate of SPTB placentas compared to AAT levels in the basal plate of term or EPTB placentas. Lower anti-protease concentrations or activity may decrease resistance to protease-catalyzed degradation of fibrinoid deposits and release of fibrinoid deposit components, such as fFN. Elevated levels of fFN have indeed been measured in cervicovaginal fluids as an early index that predicts preterm delivery [10]. Moreover, our immuno-EM and immunofluorescence images indicate that AAT and fibronectin colocalize to some extent in the extravillous fibrinoid deposit region of the human placenta. However, AAT colocalized predominantly with another fibrinoid deposit component: collagen IV.

In previous studies, AAT is associated with fibrosis that takes place in response to tissue injury or inflammation and is defined by overproduction of extracellular matrix in the connective tissue [63]. For example, the *SERPINA1* Z variant is associated with fibrotic liver disease [64]. By contrast, in pregnancy, fibrosis is linked to complications like preeclampsia [63]. Fibrosis in preeclamptic placentas is associated with stromal fibroblasts activated by the TGFβ signaling pathway, as treatment of placental fibroblasts with TGFβ1 leads to production of fibrosis-related factors like FN1 and COL4A1 [63]. Our silencing of *SERPINA1* in trophoblast culture showed that *SERPINA1* affects fibrosis-related factors like COL4A1 and FN1, which are also components of fibrinoid deposits in the placenta.

The amount of fibrinoid increases steadily throughout pregnancy. Fibrinoids have an immunosuppressive function that is mediated by binding to various types of antigens, which lowers the immunological load between mother and fetus [50, 65, 66]. Moreover, the fibrinoid barrier modulates trophoblast invasion into the uterus during early pregnancy [62, 67]. The presence of AAT in fibrinoid deposits suggests a mechanism for maintaining the stability and defense functions of fibrinoid. The lower placental expression levels of AAT in SPTB that we observed may promote inflammatory activation via the Slit–Robo pathway. Additionally, AAT is known to inhibit neutrophil elastase, which is required for normal resistance against infections, but which also can disrupt tissue homeostasis, especially in the lungs, if not adequately regulated [68, 69]. In the placenta, low anti-protease activity combined with proinflammatory activation may lead to proteolytic degradation of the fibrinoid structure, leakage of specific placental extracellular components into the cervix, and premature activation of labor.

**Fig. 10:**
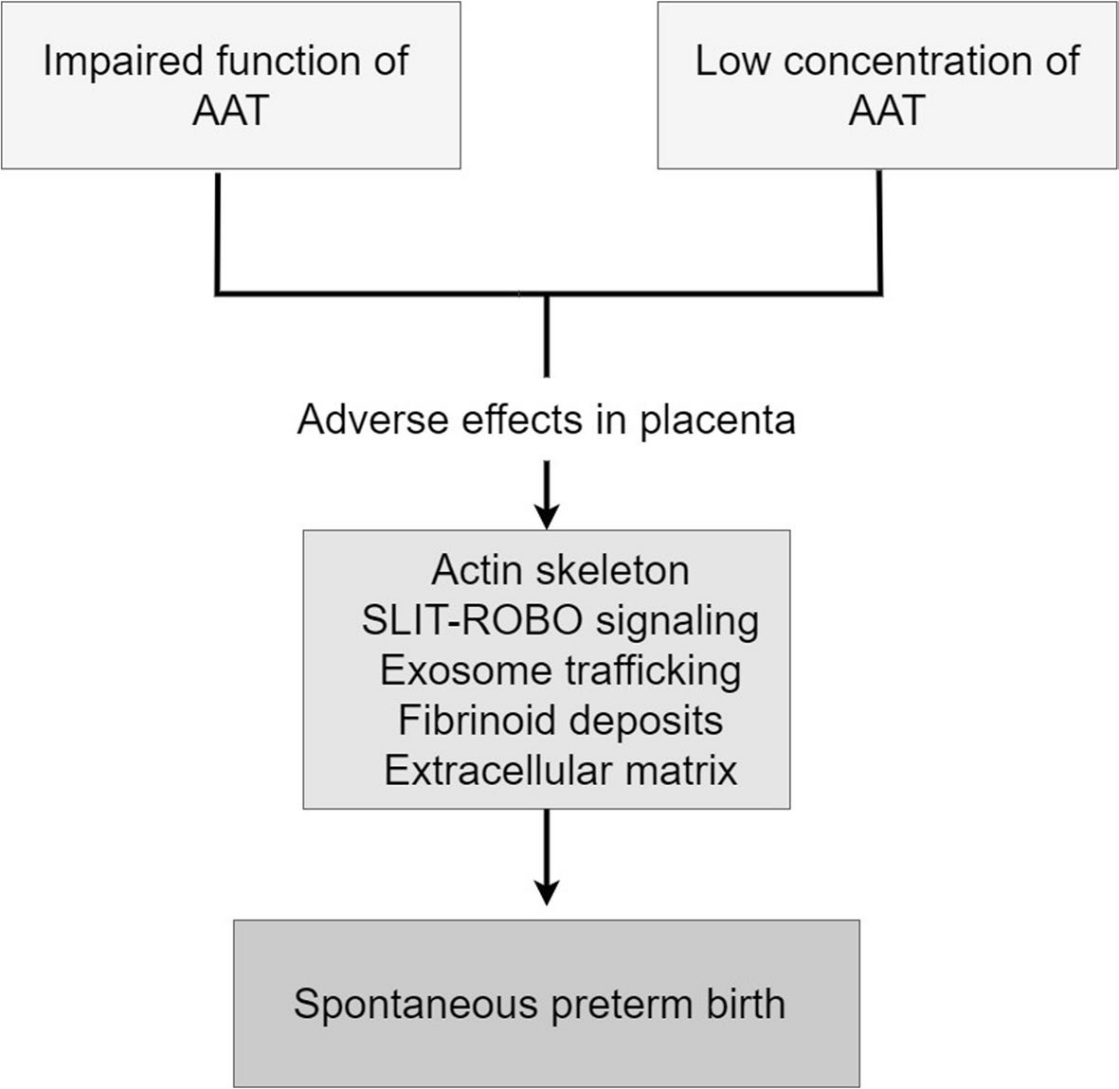
Functions of AAT in the human placenta. According to our data, we propose that AAT has multiple functions in the human placenta. AAT influence the amount and composition of fibrinoid deposit, and extracellular matrix organization. Similarly, AAT regulate actin cytoskeleton and exosome trafficking. AAT may regulate some functions through regulation of the Slit–Robo signaling pathway. Impaired function or low concentration of AAT has adverse effect in placenta and this may lead to spontaneous preterm birth

AAT seems to influence multiple pathways in placenta (Fig. 10). We propose that fibrinoid deposits and their anti-inflammatory capacity are adversely affected by low concentrations or lack of function of AAT in the placenta. This may result in degradation of fibrinoid deposits and decreased resistance against proinflammatory mediators, with consequent promotion of early labor. In addition, the amount of fibrinoid affects trophoblast invasion [62, 67]. Our EM immunohistochemistry and fluorescence colocalization analyses showed an association between AAT and fibrinoid deposit. Displacement of fFN to the cervix may reflect ongoing degradation of fibrinoid tissue. We further speculate that AAT supplementation may delay the onset and progression of premature labor, similar to prophylactic treatment of emphysema associated with damaging *SERPINA1* variants [70–72]. It has also been shown that AAT modulates TNF-α in neutrophils, such that low levels of AAT result in increased TNF-α signaling and neutrophil degranulation [73]. In the degranulation event, neutrophil elastase is released. Finally, disruption of maternal-fetal immune tolerance has been linked to chronic chorioamnionitis that often leads to spontaneous preterm labor and delivery [5]. It is interesting that experimental AAT therapy has induced immune tolerance during allograft transplantation [74]. Our hypothesis that AAT maintains fibrinoid deposits, moderates proinflammatory signaling from the placenta to the uterus, and delays premature labor/delivery remains to be proven.

## Conclusions

The focus of the study was on the role of the placenta in regulation of the length of pregnancy. We analyzed proteomes of placentas to identify proteins associated with spontaneous preterm birth. Next, the entire exons of the identified proteins were analyzed for potentially damaging variants in families with recurrent preterm births. These resulted in alpha-1 antitrypsin encoded by *SERPINA1* in association with spontaneous preterm labor. Protein and mRNA levels of alpha-1 antitrypsin/*SERPINA1* in the placenta were downregulated in spontaneous preterm births. Alpha-1 antitrypsin was expressed by villous trophoblasts in the placenta. Our immunoelectron microscopy and fluorescence colocalization analyses showed an association between alpha-1 antitrypsin and placental fibrinoid deposit with specific extracellular proteins. These fibrinoids have an immunosuppressive function that is mediated by binding to various types of antigens, which lowers the immunological load between mother and fetus. Alpha-1 antitrypsin is a protease inhibitor, and the presence of alpha-1 antitrypsin in fibrinoid deposits suggests a mechanism for maintaining the stability and defense functions of the fibrinoid. We propose that low alpha-1 antitrypsin levels result in low anti-protease activity combined with proinflammatory activation and lead to proteolytic degradation of the fibrinoid deposit structure, leakage of specific placental extracellular components into the cervix, and premature activation of labor. We further speculate that alpha-1 antitrypsin supplementation could delay the onset and progression of premature labor, similar to prophylactic treatment of emphysema associated with loss of function of alpha-1 antitrypsin in the lungs.

## Supplementary Information

**Additional file 1: FigS1.** Representative 2D gel of human placental tissue after spontaneous preterm birth. **FigS2.** Densitometric evaluation of western blot of alpha-1 antitrypsin and tubulin α-1B. **FigS3.** Proteomic differences between chorionic and basal plates of the placenta. **FigS4.** Immunoelectron microscopy images of localization of AAT and fibronectin in spontaneous term birth placenta. **FigS5.** Colocalization fluorescence image of AAT and Rab7, and AAT and CD63. **FigS6.** mRNA expression levels after gene knockdown of *SERPINA1*. **FigS7.** Gene expression changes after *SERPINA1* silencing according to RNA sequencing and qPCR. **TableS1.** Protein levels and statistical significance in comparisons of spontaneous preterm and term birth. **TableS2.** Protein levels and statistical significance in comparisons of spontaneous and elective preterm birth. **TableS3.** Protein identification. **TableS4.** Upregulated genes after *SERPINA1* silencing in HTR8/SVneo cells. **TableS5.** Downregulated genes after *SERPINA1* silencing in HTR8/SVneo cells. **TableS6.** Biological pathways affected by *SERPINA1* silencing in HTR8/SVneo cells. **TableS7.** Gene Ontology Biological Processes (GO-BP) term enrichment analysis of genes affected by *SERPINA1* silencing. **TableS8.** Gene Ontology Cellular Component (GO-CC) term enrichment analysis of genes affected by *SERPINA1* silencing. **TableS9.** Comparison of RNA sequencing and qRT-PCR of genes affected by SERPINA1 knockdown.

## Data Availability

Transcriptomic data for SERPINA1-silenced HTR8/SVneo cells were deposited in the NCBI Gene Expression Omnibus [75] and are accessible at GEO Series accession number GSE165090.

## Abbreviations

AAT: Alpha-1 antitrypsin
ACTB: Actin cytoplasmic 1
ACTC1: Actin alpha cardiac muscle 1
ACTG2: Actin gamma 2, smooth muscle
AHSG: α-2-HS-glycoprotein
ALB: Serum albumin
ANXA3: Annexin A3
ANXA4: Annexin A4
ANXA5: Annexin A5
APCS: Serum amyloid P-component
APOA1: Apolipoprotein A-I
Ba: Basal plate
BASP1: Brain acid soluble protein 1
BSA: Bovine serum albumin
CD63: CD63 molecule
cDNA: Complementary DNA
CEACAM1: Carcinoembryonic antigen-related cell adhesion molecule 1
CFB: Complement factor B
CFL1: Cofilin-1
Ch: Chorionic plate
CLIC4: Chloride intracellular channel protein 4
CLU: Clusterin
COL4A1: Collagen alpha-1 (IV) chain
CPNE1: Copine-1
CSH1/2: Chorionic somatomammotropin hormone 1/2
CYC1: Cytochrome C1
EEF2: Elongation factor 2
EFHD1: EF-hand domain-containing protein D1
EM: Electron microscope
EPTB: Elective preterm birth
ERO1L: eRO1-like protein α
EZR: Ezrin
F13A1: Coagulation factor XIII A chain
FFGC: Finnish Functional Genomics Centre
fFN: Fetal fibronectin
FGB: Fibrinogen β chain
FGG: Fibrinogen γ
FN1: Fibronectin 1
FTL: Ferritin light chain
GA: Gestational age
GO-BP: Gene ontology biological processes
GO-CC: GO cellular component
GSN: Gelsolin
GSTO1: Glutathione S-transferase ω-1
HBG1/2: Hemoglobin subunit γ-1/2
HNRNPK: Heterogeneous nuclear ribonucleoprotein K
HP: Haptoglobin
HSD17B1: Estradiol 17-β-dehydrogenase 1
HSP90B1: Endoplasmin
HSPA5: 78-kDa glucose-regulated protein
IgG: Immunoglobulin G
IL-6: Interleukin 6
IL-8: Interleukin 8
ITIH4: Inter-α trypsin inhibitor heavy chain H4
KRT18: Keratin type I cytoskeletal 18
KRT19: Keratin type I cytoskeletal 19
KRT8: Keratin type II cytoskeletal 8
LAMA2: Laminin subunit alpha 2
LAMA4: Laminin subunit alpha-4
LUM: Lumican
MAF: Minor allele frequency
mRNA: Messenger RNA
NUDT16: U8 snoRNA-decapping enzyme
PBS: Phosphate buffered saline
PPROM: Preterm premature rupture of the membranes
PRDX4: Peroxiredoxin-4
PRDX6: Peroxiredoxin-6
PTB: Preterm birth
qRT-PCR: Real-time quantitative reverse transcription PCR
Rab7: Ras-related protein 7
S100A6: Protein S100-A6
SERPINA1: Serpin family A member 1
SERPINB2: Plasminogen activator inhibitor 2
siRNA: Small interfering RNA
SLIT2: Slit guidance ligand 2
SNP: Single-nucleotide polymorphism
SPTB: Spontaneous preterm birth
STB: Spontaneous term birth
TAGLN2: Transgelin-2
TF: Serotransferrin
TGFβ: Transforming growth factor beta
TNF-α: Tumor necrosis factor alpha
TPM1: Tropomyosin α-1 chain
TPM2: Tropomyosin β chain
VIM: Vimentin
WARS: Tryptophan-tRNA ligase
WES: Whole exome sequencing
