# Supplementary material for: Human placental proteomics and exon variant studies link AAT/SERPINA1 with spontaneous preterm birth
Source: BMC Med. 2022 Apr 28;20:141. doi: 10.1186/s12916-022-02339-8 (PMC9047282; doi:10.1186/s12916-022-02339-8)
Supplement: Supplementary file 1 — Additional file 1: FigS1. Representative 2D gel of human placental tissue after spontaneous preterm birth. FigS2. Densitometric evaluation of western blot of alpha-1 antitrypsin and tubulin α-1B. FigS3. Proteomic differences between chorionic and basal plates of the placenta. FigS4. Immunoelectron microscopy images of localization of AAT and fibronectin in spontaneous term birth placenta. FigS5. Colocalization fluorescence image of AAT and Rab7, and AAT and CD63. FigS6. mRNA expression levels after gene knockdown of SERPINA1. FigS7. Gene expression changes after SERPINA1 silencing according to RNA sequencing and qPCR. TableS1. Protein levels and statistical significance in comparisons of spontaneous preterm and term birth. TableS2. Protein levels and statistical significance in comparisons of spontaneous and elective preterm birth. TableS3. Protein identification. TableS4. Upregulated genes after SERPINA1 silencing in HTR8/SVneo cells. TableS5. Downregulated genes after SERPINA1 silencing in HTR8/SVneo cells. TableS6. Biological pathways affected by SERPINA1 silencing in HTR8/SVneo cells. TableS7. Gene Ontology Biological Processes (GO-BP) term enrichment analysis of genes affected by SERPINA1 silencing. TableS8. Gene Ontology Cellular Component (GO-CC) term enrichment analysis of genes affected by SERPINA1 silencing. TableS9. Comparison of RNA sequencing and qRT-PCR of genes affected by SERPINA1 knockdown. [file 12916_2022_2339_MOESM1_ESM.docx]

**Additional file**


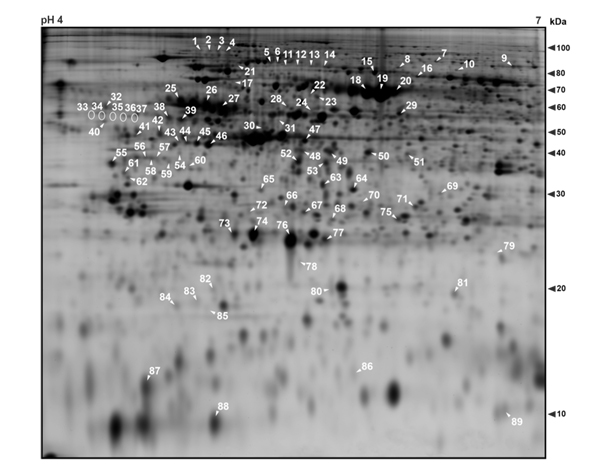


**Fig S1. Representative 2D gel of human placental tissue after spontaneous preterm birth.** Placental proteins (50 μg) collected from basal plate of the placenta were labeled with Cy5 (minimal DIGE) and separated by isoelectic focusing (pH 4–7, 24 cm) and SDS-PAGE. Significantly changed spots are indicated.


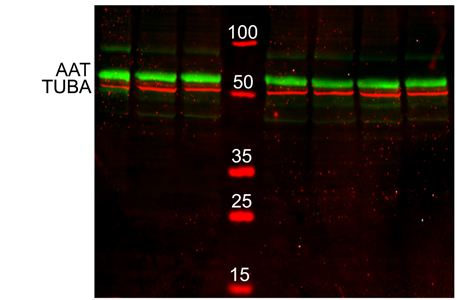


**Fig S2. Densitometric evaluation of western blot of alpha-1 antitrypsin (AAT, green) and tubulin α-1B (TUBA, red).** In this western blot, placental samples from chorionic plate of SPTB (*n* = 6) are shown; sample on the left is the run calibrator. Sizes (kDa) of the protein ladder are also shown.


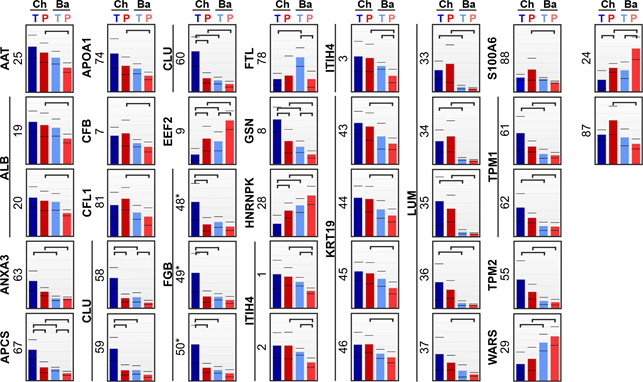


**Fig S3. Proteomic differences between chorionic (Ch) and basal (Ba) plates of the placenta.** Samples were collected after spontaneous preterm (P) and term (T) birth. Exact positions of spots are presented in Fig S1. Corresponding changes in protein levels are shown in the expression profile, and statistically significant changes (p < 0.05) are indicated by horizontal brackets. Horizontal brackets identify the compared groups. Spots corresponding to the same protein are clustered with a vertical line, and protein fragments are indicated with asterisks.


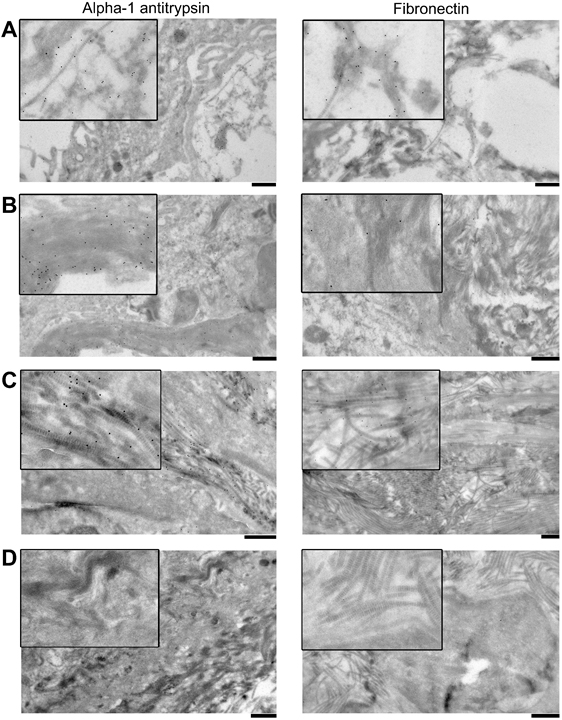


**Fig S4. Immunoelectron microscopy images of localization of AAT and fibronectin in spontaneous term birth placenta.** Samples were from the basal plate (maternal side) of the placenta and were immunostained with anti‐human AAT (left panel) and anti-human fibronectin (right panel) antibodies (A–C). Bound antibodies were labeled by incubation with protein A–conjugated gold particles (black dots). In controls, primary antibodies were omitted (D). Upper left corner image is the zoomed-in view of a region of the image located underneath. Scale bar: 0.5 μm.


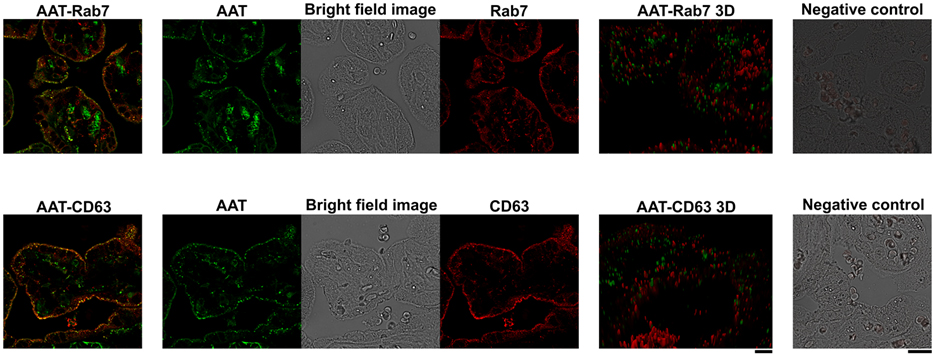


**Fig S5. Colocalization fluorescence image of AAT (green) and Rab7 (red) (top row) and AAT (green) and CD63 (red) (bottom row).** Rab7 is known as a late endosome or lysosome marker, and CD63 is an exosome marker. Middle columns are separate green channel, red channel, and brightfield images, which are shown merged in left column. Panels in right column show negative controls treated the same way as samples but with primary antibody omitted. Objective used was HC PL APO CS2 63×/1.40 OIL. Scale bar: 20 µm, except for 3D images (5 µm).


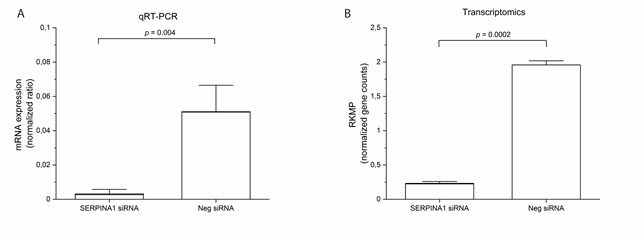


**Fig S6. mRNA expression levels after gene knockdown of *SERPINA1***. *SERPINA1* was post-transcriptionally knocked down with siRNA in the human placental trophoblast continuous cell line HTR8/SVneo. mRNA levels of cells in which *SERPINA1* was knocked down were compared with mRNA levels from siRNA negative–treated control cells. Expression levels of genes were first determined by qRT-PCR (levels normalized against housekeeping gene *CYC1* mRNA levels). According to qRT-PCR, *SERPINA1* was 94% silenced (A). Gene expression levels were also determined with high-throughput RNA sequencing. Reads per kilobase of exon per million reads mapped (RKPM) is a normalized gene count value determined in transcriptomic analysis. According to transcriptomics data, *SERPINA1* was 89% silenced (B). Columns represent median, mean, and standard deviation values.


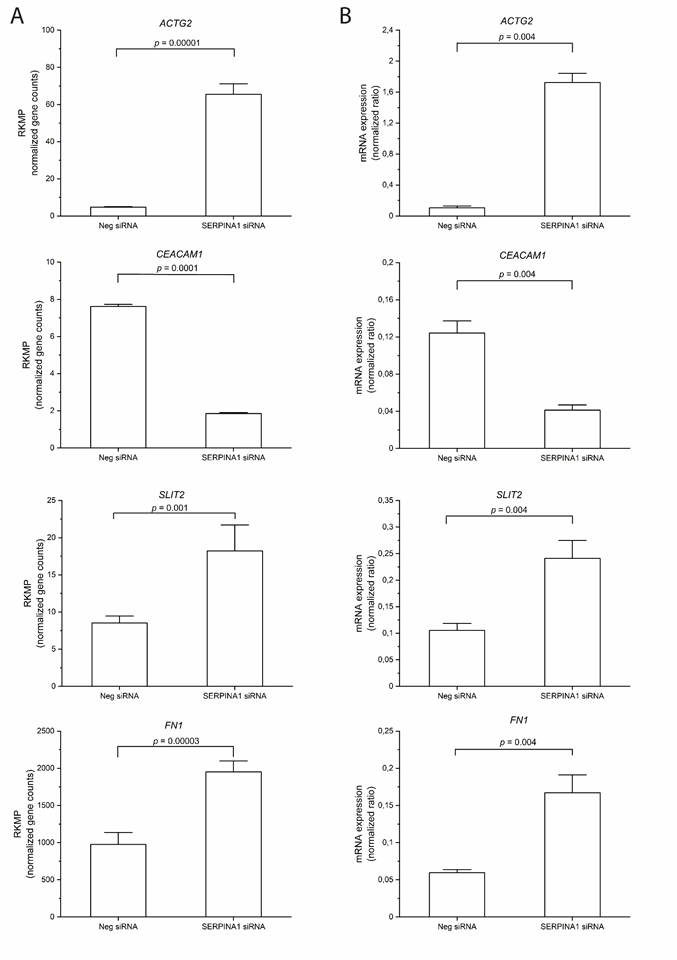


**Fig S7. Gene expression changes after *SERPINA1* silencing according to RNA sequencing (A) and qPCR (B).** (A) Four genes affected by *SERPINA1* silencing according to RNA sequencing. (B) Results of the RNA sequencing were verified by qRT-PCR. Expression changes for *ACTG2, CAECAM1, SLIT2,* and *FN1* after *SERPINA1* silencing were measured with qRT-PCR. *SERPINA1* was post-transcriptionally silenced in HTR8/SVneo cell line by siRNA. mRNA levels of selected genes compared between *SERPINA1*-silenced cells and untreated control cells. All mRNA levels are normalized against mRNA levels of housekeeping gene *CYC1*. Columns represent median and standard deviation values of the sample groups.

**Table S1. Protein levels and statistical significance in comparisons of spontaneous preterm and term birth.**

| Spot | Protein | UniProt-KB | Description | Mean normalized volumes | | | | *t*-test | | | |
| --- | --- | --- | --- | --- | --- | --- | --- | --- | --- | --- | --- |
|  |  |  |  | **Ba** | | **Ch** | | **P vs. T** | | **Ba vs. Ch** | |
|  |  |  |  | **P** | **T** | **P** | **T** | **Ba** | **Ch** | **P** | **T** |
| Spontaneous preterm (P) vs. term (T) birth | | | | | | | | | | | |
|  |  |  |  |  |  |  |  |  |  |  |  |
| 26  27  38  39 | AAT  AAT  AAT  AAT | P01009  P01009  P01009  P01009 | α-1-antitrypsin (isoform 1)  α-1-antitrypsin (isoform 1)  α-1-antitrypsin (isoform 1,2)  α-1-antitrypsin (isoform 1,2) | 189  198  149  165 | 284  322  299  301 | 284  292  86  90 | 362  424  206  194 | 0.0204  0.0024  0.2030  0.2153 | 0.1557  0.0269  0.0126  0.0263 | 0.0189  0.0155  0.1801  0.1384 | 0.1638  0.0719  0.4117  0.3131 |
| 85* | ACTB | P60709 | Actin, cytoplasmic 1 (fragment) | 432 | 174 | 356 | 269 | 0.0190 | 0.5992 | 0.6385 | 0.2713 |
| 18 | ALB | P02768 | Serum albumin | 157 | 238 | 259 | 273 | 0.0220 | 0.8235 | 0.0929 | 0.4041 |
| 84* | ANXA5 | P08758 | Annexin A5 (fragment) | 434 | 198 | 287 | 213 | 0.0173 | 0.4127 | 0.1929 | 0.7853 |
| 67 | APCS | P02743 | Serum amyloid P-component | 179 | 290 | 330 | 799 | 0.0065 | 0.0051 | 0.0462 | 0.0014 |
| 73 | APOA1 | P02647 | Apolipoprotein A-I | 159 | 243 | 260 | 374 | 0.0206 | 0.1900 | 0.0704 | 0.0955 |
| 58  59  60 | CLU  CLU  CLU | P10909  P10909  P10909 | Clusterin (isoform 3)  Clusterin (isoform 3)  Clusterin (isoform 3) | 139  161  190 | 274  282  284 | 254  282  351 | 784  839  1051 | 0.0484  0.0748  0.0983 | 0.0023  0.0037  0.0031 | 0.0592  0.1161  0.0451 | 0.0033  0.0031  0.0014 |
| 23 | CPNE1 | Q99829 | Copine-1 | 333 | 170 | 288 | 178 | 0.0019 | 0.0136 | 0.2367 | 0.8243 |
| 9 | EEF2 | P13639 | Elongation factor 2 | 392 | 202 | 227 | 87 | 0.0052 | 0.0262 | 0.0218 | 0.0281 |
| 48*  49*  50*  51* | FGB  FGB  FGB  FGB | P02675  P02675  P02675  P02675 | Fibrinogen ß chain (C-terminal fragment)  Fibrinogen ß chain (C-terminal fragment)  Fibrinogen ß chain (C-terminal fragment)  Fibrinogen ß chain (C-terminal fragment) | 256  237  218  279 | 368  331  315  376 | 313  345  379  277 | 902  1050  1076  681 | 0.1931  0.1668  0.1631  0.3729 | 0.0287  0.0252  0.0226  0.0288 | 0.5489  0.2468  0.0576  0.9906 | 0.0394  0.0202  0.0139  0.0070 |
| 11  12  13  14 | FGG  FGG  FGG  FGG | P02679  P02679  P02679  P02679 | Fibrinogen γ (isoform γ-B,A)  Fibrinogen γ (isoform γ-B,A)  Fibrinogen γ (isoform γ-B,A)  Fibrinogen γ (isoform γ-B,A) | 207  208  213  215 | 412  381  359  337 | 125  152  189  210 | 449  519  550  561 | 0.3362  0.3428  0.3384  0.3252 | 0.0186  0.0175  0.0236  0.0426 | 0.3929  0.5692  0.7997  0.9414 | 0.8667  0.4918  0.2925  0.2238 |
| 78 | FTL | P02792 | Ferritin light chain | 149 | 407 | 192 | 148 | 0.0025 | 0.6938 | 0.7113 | 0.0011 |
| 8 | GSN | P06396 | Gelsolin (isoform 1) | 156 | 279 | 373 | 724 | 0.0352 | 0.0181 | 0.0344 | 0.0012 |
| 89 | HBG1/2 | P69891/2 | Hemoglobin subunit γ-1/2 | 538 | 211 | 310 | 224 | 0.0404 | 0.5254 | 0.7900 | 0.8416 |
| 28 | HNRNPK | P61978 |  | 362 | 272 | 229 | 111 | 0.0786 | 0.0262 | 0.0153 | 0.0042 |
| 17 | HSPA5 | P11021 | 78 kDa glucose-regulated protein | 561 | 206 | 288 | 111 | 0.0295 | 0.0250 | 0.0897 | 0.2714 |
| 1  2  3  4 | ITIH4  ITIH4  ITIH4  ITIH4 | Q14624  Q14624  Q14624  Q14624 | Inter-α-trypsin inhibitor heavy chain H4 (isoform 1)  Inter-α-trypsin inhibitor heavy chain H4 (isoform 1)  Inter-α-trypsin inhibitor heavy chain H4 (isoform 1)  Inter-α-trypsin inhibitor heavy chain H4 (isoform 1) | 195  189  181  191 | 299  300  295  298 | 359  364  388  351 | 387  365  407  414 | 0.0031  0.0017  0.0150  0.0047 | 0.7579  0.9870  0.8591  0.5537 | 0.0133  0.0364  0.0429  0.0827 | 0.2784  0.3048  0.1890  0.1424 |
| 31  30  54*  57* | KRT8  KRT8  KRT8  KRT8 | P05787  P05787  P05787  P05787 | Keratin, type II cyto-skeletal 8 (isoform 1,2)  Keratin, type II cyto-skeletal 8 (isoform 1,2)  Keratin, type II cyto-skeletal 8 (isoform 1,2; fragment)  Keratin, type II cyto-skeletal 8 (isoform 1,2; fragment) | 190  203  81  90 | 293  272  308  315 | 172  218  96  140 | 427  338  481  658 | 0.3283  0.1649  0.2395  0.2407 | 0.0071  0.0332  0.0022  0.0019 | 0.6464  0.7139  0.6212  0.2164 | 0.2798  0.2380  0.4139  0.1416 |
| 5  6 | KRT18  KRT18 | P05783  P05783 | Keratin, type I cytoskeletal 18  Keratin, type I cytoskeletal 18 | 172  156 | 316  324 | 130  119 | 264  262 | 0.3555  0.3611 | 0.0119  0.0194 | 0.3389  0.5113 | 0.7346  0.7294 |
| 56* | KRT19 | P08727 | Keratin, type I cytoskeletal 19 (C-terminal fragment) | 139 | 278 | 188 | 428 | 0.1033 | 0.0023 | 0.2189 | 0.1276 |
| 79 | NUDT16 | Q96DE0 | U8 snoRNA-decapping enzyme (isoform 1) | 866 | 181 | 532 | 209 | 0.1953 | 0.0396 | 0.5200 | 0.6976 |
| 10 | TF | P02787 | Serotransferrin | 336 | 172 | 347 | 143 | 0.0087 | 0.0222 | 0.7722 | 0.4274 |
| 80 | TAGLN2 | P37802 | Transgelin-2 (isoform 1,2) | 153 | 267 | 196 | 375 | 0.0029 | 0.0053 | 0.2425 | 0.0405 |
| 41 | VIM | P08670 | Vimentin | 184 | 311 | 213 | 375 | 0.0048 | 0.0271 | 0.1341 | 0.3760 |
| 24 | - | - | - | 384 | 188 | 210 | 105 | 0.0059 | 0.0269 | 0.0029 | 0.1201 |
| Basal plate (Ba) vs. chorionic plate (Ch) | | | | | | | | | | | |
| 25 | AAT | P01009 | α-1-antitrypsin (isoform 1) | 181 | 256 | 292 | 337 | 0.0185 | 0.3928 | 0.0111 | 0.0924 |
| 19  20 | ALB  ALB | P02768  P02768 | Serum albumin (isoform 1)  Serum albumin (isoform 1) | 187  170 | 267  252 | 288  260 | 310  285 | 0.0308  0.0188 | 0.6738  0.6041 | 0.0344  0.0224 | 0.3574  0.4728 |
| 63 | ANXA3 | P12429 | Annexin A3 | 251 | 251 | 433 | 709 | 0.9944 | 0.0515 | 0.0049 | 0.0031 |
| 67 | APCS | P02743 | Serum amyloid P-component | 179 | 290 | 330 | 799 | 0.0065 | 0.0051 | 0.0462 | 0.0014 |
| 74 | APOA1 | P02647 | Apolipoprotein A-I | 185 | 269 | 302 | 449 | 0.0293 | 0.1371 | 0.0414 | 0.0548 |
| 7 | CFB | P00751 | Complement factor B (isoform1) | 177 | 216 | 321 | 294 | 0.2058 | 0.7171 | 0.0409 | 0.0941 |
| 81 | CFL1 | P23528 | Cofilin-1 | 233 | 282 | 443 | 371 | 0.4253 | 0.4361 | 0.0178 | 0.2717 |
| 58  59  60 | CLU  CLU  CLU | P10909  P10909  P10909 | Clusterin (isoform 3)  Clusterin (isoform 3)  Clusterin (isoform 3) | 139  161  190 | 274  282  284 | 254  282  351 | 784  839  1051 | 0.0484  0.0748  0.0983 | 0.0023  0.0037  0.0031 | 0.0592  0.1161  0.0451 | 0.0033  0.0031  0.0014 |
| 9 | EEF2 | P13639 | Elongation factor 2 | 392 | 202 | 227 | 87 | 0.0052 | 0.0262 | 0.0218 | 0.0281 |
| 48*  49*  50* | FGB  FGB  FGB | P02675  P02675  P02675 | Fibrinogen ß chain (C-terminal fragment)  Fibrinogen ß chain (C-terminal fragment)  Fibrinogen ß chain (C-terminal fragment) | 256  237  218 | 368  331  315 | 313  345  379 | 902  1050  1076 | 0.1931  0.1668  0.1631 | 0.0287  0.0252  0.0226 | 0.5489  0.2468  0.0576 | 0.0394  0.0202  0.0139 |
| 78 | FTL | P02792 | Ferritin light chain | 149 | 407 | 192 | 148 | 0.0025 | 0.6938 | 0.7113 | 0.0011 |
| 8 | GSN | P06396 | Gelsolin (isoform 1) | 156 | 279 | 373 | 724 | 0.0352 | 0.0181 | 0.0344 | 0.0012 |
| 28 | HNRNPK | P61978 | Heterogeneous nuclear ribonucleoprotein K (isoform 1-3) | 362 | 272 | 229 | 111 | 0.0786 | 0.0262 | 0.0153 | 0.0042 |
| 1  2  3 | ITIH4  ITIH4  ITIH4 | Q14624  Q14624  Q14624 | Inter-α-trypsin inhibitor heavy chain H4 (isoform 1)  Inter-α-trypsin inhibitor heavy chain H4 (isoform 1)  Inter-α-trypsin inhibitor heavy chain H4 (isoform 1) | 195  189  181 | 299  300  295 | 359  364  388 | 387  365  407 | 0.0031  0.0017  0.0150 | 0.7579  0.9870  0.8591 | 0.0133  0.0364  0.0429 | 0.2784  0.3048  0.1889 |
| 43  44  45  46 | KRT19  KRT19  KRT19  KRT19 | P08727  P08727  P08727  P08727 | Keratin, type I cytoskeletal 19  Keratin, type I cytoskeletal 19  Keratin, type I cytoskeletal 19  Keratin, type I cytoskeletal 19 | 181  181  178  206 | 246  234  259  237 | 332  325  309  324 | 365  336  325  319 | 0.2715  0.2435  0.0625  0.3149 | 0.6528  0.8763  0.7873  0.9376 | 0.0269  0.0286  0.0222  0.0168 | 0.1168  0.1094  0.2020  0.1475 |
| 33  34  35  36  37 | LUM  LUM  LUM  LUM  LUM | P51884  P51884  P51884  P51884  P51884 | Lumican  Lumican  Lumican  Lumican  Lumican | 157  160  131  150  158 | 222  226  207  220  248 | 1453  1228  1241  829  468 | 1112  1021  1571  1152  692 | 0.1820  0.0832  0.1313  0.1729  0.2503 | 0.5037  0.5964  0.5628  0.4425  0.3109 | 0.0181  0.0065  0.0021  0.0032  0.0049 | 0.0020  0.0047  0.0182  0.0294  0.0557 |
| 88 | S100A6 | P06703 | Protein S100-A6 | 246 | 332 | 588 | 373 | 0.0074 | 0.2245 | 0.0120 | 0.3506 |
| 61 | TPM1 | P09493 | Tropomyosin α-1 chain (isoform 2,3,4) | 264 | 304 | 524 | 920 | 0.5969 | 0.1224 | 0.0103 | 0.0237 |
| 62 | TPM1 | P09493 | Tropomyosin α-1 chain (isoform 1) | 286 | 294 | 543 | 854 | 0.9168 | 0.1380 | 0.0125 | 0.0143 |
| 55 | TPM2 | P07951 | Tropomyosin ß chain (isoform 2) | 270 | 325 | 737 | 1272 | 0.5605 | 0.2094 | 0.0134 | 0.0311 |
| 29 | WARS | P23381 | Tryptophan-tRNA ligase, cytoplasmic (isoform 1) | 332 | 283 | 163 | 124 | 0.2719 | 0.4991 | 0.0065 | 0.0083 |
| 24 | - | - | - | 384 | 188 | 210 | 105 | 0.0059 | 0.0269 | 0.0029 | 0.1201 |
| 87 | - | - | - | 240 | 316 | 518 | 348 | 0.1716 | 0.0570 | 0.0054 | 0.5373 |

Spot numbers and identified proteins according to Fig S1 and Tables 1 and 3 are shown. Protein levels are represented by mean normalized spot volumes for spontaneous preterm (P) and term (T) birth in samples collected from basal (Ba) or chorionic (Ch) plates of the placenta. Statistical significance according to Student’s *t*-test with *p* < 0.05 as the cut-off is indicated for comparisons of P vs. T and Ba vs. Ch in the corresponding subgroups Ba and Ch or P and T.

**Table S2. Protein levels and statistical significance in comparisons of spontaneous and elective preterm birth.**

| Spot | Protein | UniProt-KB | Description | Mean normalized volumes | | | | *t*-test | |
| --- | --- | --- | --- | --- | --- | --- | --- | --- | --- |
|  |  |  |  | **Ba** | | **Ch** | | **S vs. E** | |
|  |  |  |  | **S** | **E** | **S** | **E** | **Ba** | **Ch** |
| 26 | AAT | P01009 | α-1-antitrypsin (isoform 1 or 2) | 190 | 292 | 285 | 311 | 0.0108 | 0.5751 |
| 32 | AHSG | P02765 | α-2-HS-glycoprotein (inactive protein with propeptide) | 223 | 395 | 360 | 414 | 0.0083 | 0.5614 |
| 18  19  20 | ALB  ALB  ALB | P02768  P02768  P02768 | Serum albumin (isoform 1)  Serum albumin (isoform 1)  Serum albumin (isoform 1) | 157  187  170 | 238  283  267 | 260  288  260 | 214  250  228 | 0.0363  0.0433  0.0245 | 0.4871  0.4541  0.4761 |
| 64 | ANXA4 | P09525 | Annexin A4 (isoform 1) | 245 | 485 | 260 | 529 | 0.0219 | 0.0158 |
| 83 | ANXA5 | P08758 | Annexin A5 | 417 | 201 | 232 | 147 | 0.0369 | 0.0748 |
| 40 | BASP1 | P80723 | Brain acid soluble protein 1 (isoform 1) | 183 | 591 | 192 | 464 | 0.0409 | 0.0025 |
| 81 | CFL1 | P23528 | Cofilin-1 | 233 | 565 | 442 | 511 | 0.0197 | 0.5289 |
| 71 | CLIC3 | O95833 | Chloride intracellular channel protein 3 | 269 | 455 | 252 | 440 | 0.0988 | 0.0425 |
| 66 | CLIC4 | Q9Y696 | Chloride intracellular channel protein 4 | 280 | 437 | 342 | 327 | 0.0234 | 0.8279 |
| 76  77  86* | CSH1/2  CSH1/2  CSH1/2 | P0DML2/3  P0DML2/3  P0DML2/3 | Chorionic somatomammotropin hormone 1/2 (isoform 1)  Chorionic somatomammotropin hormone 1/2 (isoform 1)  Chorionic somatomammotropin hormone 1/2 (isoform 1, fragment) | 347  319  414 | 111  153  134 | 290  285  255 | 90  131  123 | 0.0002  0.0005  0.0216 | 0.0113  0.0513  0.0613 |
| 65 | EFHD1 | Q9BUP0 | EF-hand domain-containing protein D1 (isoform 1) | 273 | 679 | 288 | 578 | 0.0259 | 0.0219 |
| 22 | ERO1L | Q96HE7 | ERO1-like protein α | 244 | 677 | 316 | 526 | 0.0091 | 0.0153 |
| 16 | EZR | P15311 | Ezrin | 178 | 479 | 215 | 432 | 0.0103 | 0.0039 |
| 15 | F13A1 | P00488 | Coagulation factor XIII A chain (inactive protein with propeptide) | 335 | 169 | 297 | 142 | 0.0151 | 0.0067 |
| 70 | GSTO1 | P78417 | Glutathione S-transferase ω-1 (isoform 1) | 198 | 398 | 219 | 361 | 0.0399 | 0.1735 |
| 42 | HP | P00738 | Haptoglobin (isoform 1 or 2) | 210 | 143 | 341 | 156 | 0.2036 | 0.0467 |
| 52  53 | HSD17B1  HSD17B1 | P14061  P14061 | Estradiol 17-β-dehydrogenase 1  Estradiol 17-β-dehydrogenase 1 | 278  293 | 169  172 | 217  235 | 161  165 | 0.0261  0.0261 | 0.3674  0.2885 |
| 17 | HSPA5 | P11021 | 78 kDa glucose-regulated protein | 589 | 212 | 301 | 151 | 0.0205 | 0.0828 |
| 21 | HSP90B1 | P14625 | Endoplasmin | 288 | 155 | 189 | 100 | 0.0137 | 0.0509 |
| 43  44  45  46  56* | KRT19  KRT19  KRT19  KRT19  KRT19 | P08727  P08727  P08727  P08727  P08727 | Keratin, type I cytoskeletal 19  Keratin, type I cytoskeletal 19  Keratin, type I cytoskeletal 19  Keratin, type I cytoskeletal 19  Keratin, type I cytoskeletal 19 (C-terminal fragment) | 181  182  178  206  138 | 315  319  285  338  235 | 335  326  310  325  189 | 373  383  377  395  197 | 0.0377  0.0306  0.0521  0.0310  0.0349 | 0.6811  0.5030  0.3645  0.2514  0.8561 |
| 34 | LUM | P51884 | Lumican | 160 | 290 | 1249 | 864 | 0.0039 | 0.4288 |
| 68 | PRDX4 | Q13162 | Peroxiredoxin-4 | 266 | 413 | 218 | 265 | 0.0341 | 0.1178 |
| 75 | PRDX6 | P30041 | Peroxiredoxin-6 | 198 | 336 | 205 | 339 | 0.0306 | 0.0100 |
| 47 | SERPINB2 | P05120 | Plasminogen activator inhibitor 2 | 296 | 151 | 206 | 133 | 0.0096 | 0.1329 |
| 88 | S100A6 | P06703 | Protein S100-A6 | 1288 | 735 | 588 | 822 | 0.0001 | 0.5718 |
| 41 | VIM | P08670 | Vimentin | 184 | 284 | 213 | 204 | 0.0463 | 0.6486 |
| 29 | WARS | P23381 | Tryptophan-tRNA ligase (isoform 1 or 2) | 332 | 201 | 162 | 165 | 0.0045 | 0.9492 |
| 24 | - | - | - | 398 | 166 | 212 | 152 | 0.0064 | 0.0166 |
| 69 | - | - | - | 185 | 465 | 113 | 444 | 0.0773 | 0.0329 |
| 72 | - | - | - | 281 | 709 | 238 | 429 | 0.0289 | 0.0625 |
| 82 | - | - | - | 220 | 525 | 201 | 502 | 0.0331 | 0.0318 |

Spot numbers and identified proteins according to Fig S1 and Table 2 are shown. Protein levels are represented by mean normalized spot volumes for spontaneous (S) and elective (E) preterm birth in samples collected from basal (Ba) or chorionic (Ch) plates of the placenta. Statistical significance according to Student’s *t*-test with *p* < 0.05 as the cut-off is indicated for comparisons of S vs. E and Ba vs. Ch in the corresponding subgroups Ba and Ch or S and E.

**Table S3. Protein identification.**

| Spot | Protein | UniProt-KB | Description | Amino acids | Amino acids covered by MS | Score  (MS, MSMS) | SC (P)  (MS, MSMS) |
| --- | --- | --- | --- | --- | --- | --- | --- |
| 25  26  27  38  39 | AAT  AAT  AAT  AAT  AAT | P01009  P01009  P01009  P01009  P01009 | α-1-antitrypsin (isoform 1)  α-1-antitrypsin (isoform 1)  α-1-antitrypsin (isoform 1)  α-1-antitrypsin (isoform 1, 2)  α-1-antitrypsin (isoform 1, 2) | 418  418  418  418  418 | 35-404  35-404  35-306  35-411  35-404 | 682.0, 113.0  544.0, 105.0  144.0, 75.0  822.0, 148.0  813.0, 165.0 | 47.0(20), 22.6(7)  34.5(14), 20.6(7)  26.2(9), 4.9(1)  46.7(19), 20.6(7)  51.8(21), 18.5(7) |
| 85* | ACTB | P60709 | Actin, cytoplasmic 1 (fragment) | 375 | 10-113 | 147.0, 83.0 | 45.7(6), 17.7(2) |
| 32 | AHSG | P02765 | α-2-HS-glycoprotein (inactive protein with propeptide) | 367 | 104-338 | 66.6, 200.7 | 21.2(6), 10.1(2) |
| 18  19  20 | ALB  ALB  ALB | P02768  P02768  P02768 | Serum albumin  Serum albumin (isoform 1)  Serum albumin (isoform 1) | 609  609  609 | 35-581  1-581  35-581 | 732.0, 115.0  723.0, 127.0  688.0, 96.0 | 61.3(35), 16.6(8)  56.6(35), 15.8(7)  56.1(32), 15.9(7) |
| 63 | ANXA3 | P12429 | Annexin A3 | 323 | 10-317 | 487.0, 484.5 | 52.3(19), 47.1(16) |
| 64 | ANXA4 | P09525 | Annexin A4 (isoform 1) | 319 | 10-300 | 665.0, 86.0 | 60.1(24), 28.7(9) |
| 83  84* | ANXA5  ANXA5 | P08758  P08758 | Annexin A5  Annexin A5 (fragment) | 320  320 | 30-301  7-286 | 169.0, 28.0  276.0, 112.0 | 36.7(12), 3.1(2)  39.2(11), 11.6(3) |
| 67 | APCS | P02743 | Serum amyloid P-component | 223 | 27-165 | 466.0, 134.0 | 34.8(11), 23.5(4) |
| 73  74 | APOA1  APOA1 | P02647  P02647 | Apolipoprotein A-I  Apolipoprotein A-I | 267  267 | 25-239  25-262 | 430.0, 703.2  506.0, 821.7 | 43.2(15), 43.2(15)  70.8(23), 56.0(18) |
| 40 | BASP1 | P80723 | Brain acid soluble protein 1 (isoform 1) | 227 | 39-227 | 244.0, 155.0 | 48.0(5), 25.1(2) |
| 7 | CFB | P00751 | Complement factor B (isoform1) | 764 | 51-764 | 116.0, 42.0 | 27.2(17), 3.1(1) |
| 81 | CFL1 | P23528 | Cofilin-1 | 166 | 46-166 | 242.0, 94.0 | 61.7(7), 16.8(2) |
| 71 | CLIC3 | O95833 | Chloride intracellular channel protein 3 | 236 | 12-236 | 629.0, 108.0 | 81.4(16), 58.9(9) |
| 66 | CLIC4 | Q9Y696 | Chloride intracellular channel protein 4 | 253 | 2-253 | 119.0, 3.0 | 58.0(12), 14.7(1) |
| 58  59  60 | CLU  CLU  CLU | P10909  P10909  P10909 | Clusterin (isoform 3)  Clusterin (isoform 3)  Clusterin (isoform 3) | 274  274  274 | 8-269  8-269  8-269 | 433.0, 156.0  209.0, 209.0  231.0, 86.0 | 36.5(10), 18.6(3)  31.4(7),10.2(2)  31.8(8), 12.4(2) |
| 23 | CPNE1 | Q99829 | Copine-1 | 537 | 385-514 | 138.0, 43.0 | 22.3(4), 12.6(2) |
| 76  77  86* | CSH1/2  CSH1/2  CSH1/2 | P0DML2/3  P0DML2/3  P0DML2/3 | Chorionic somatomammotropin hormone 1/2 (isoform 1)  Chorionic somatomammotropin hormone 1/2 (isoform 1) Chorionic somatomammotropin hormone 1/2 (isoform 1, fragment) | 217^1^  217^1^  217^1^ | 27-204^1^  27-204^1^  51-204^1^ | 448.0, 83.0  330.0, 69.0  113.0, 25.0 | 88.0(15), 23.0(4)  62.8(11), 15.7(3)  53.9(8), 18.6(2) |
| 9 | EEF2 | P13639 | Elongation factor 2 | 858 | 367-801 | 117.0, 42.0 | 23.8(11), 3.1(1) |
| 65 | EFHD1 | Q9BUP0 | EF-hand domain-containing protein D1 (isoform 1) | 239 | 16-216 | 327.0, 97.0 | 45.2(9), 17.2(3) |
| 22 | ERO1L | Q96HE7 | ERO1-like protein α | 468 | 56-468 | 225.0, 73.0 | 35.7(17), 5.2(2) |
| 16 | EZR | P15311 | Ezrin | 586 | 2-586 | 505.0, 72.0 | 51.7(34), 9.2(6) |
| 48*  49*  50*  51* | FGB  FGB  FGB  FGB | P02675  P02675  P02675  P02675 | Fibrinogen ß chain (C-terminal fragment)  Fibrinogen ß chain (C-terminal fragment)  Fibrinogen ß chain (C-terminal fragment)  Fibrinogen ß chain (C-terminal fragment) | 491  491  491  491 | 179-491  179-491  164-491  164-491 | 725.0, 180.0  594.0, 104.0  1060.0, 162.0  222.0, 76.0 | 57.8(16), 21.1(6)  56.4(17), 25.3(6)  75.0(27), 28.0(8)  54.3(15), 6.4(1) |
| 11  12  13  14 | FGG  FGG  FGG  FGG | P02679  P02679  P02679  P02679 | Fibrinogen γ (isoform γ-B,A)  Fibrinogen γ (isoform γ-B,A)  Fibrinogen γ (isoform γ-B,A)  Fibrinogen γ (isoform γ-B,A) | 453  453  453  453 | 41-417  32-417  115-417  32-417 | 597.0, 113.0  559.0, 121.0  131.0, 51.0  554.0, 101.0 | 60.7(18), 16.8(6)  49.0(15), 14.6(5)  27.9(9), 7.5(2)  62.0(20), 14.6(5) |
| 78 | FTL | P02792 | Ferritin light chain | 175 | 41-169 | 263.0, 122.0 | 62.2(8), 19.6(2) |
| 15 | F13A1 | P00488 | Coagulation factor XIII A chain (inactive protein with propeptide) | 732 | 14-728 | 214.0,59.0 | 34.1(20), 4.7(2) |
| 8 | GSN | P06396 | Gelsolin (isoform 1) | 755 | 33-740 | 227.0, 73.0 | 23.8(16), 1.4(1) |
| 70 | GSTO1 | P78417 | Glutathione S-transferase ω-1 (isoform 1) | 241 | 12-220 | 138.0, 72.0 | 29.6(9), 5.8(1) |
| 89 | HBG1/2 | P69891/2 | Hemoglobin subunit γ-1/2 | 147 | 2-105 | 290.0, 94.0 | 92.4(9), 30.5(3) |
| 28 | HNRNPK | P61978 | Heterogeneous nuclear ribonucleoprotein K (isoform 1-3) | 463 | 36-316 | 52.6, 39.0 | 8.4(3), 2.4(1) |
| 42 | HP | P00738 | Haptoglobin (isoform 1, 2) | 406^1^ | 262-345 | 80.2, 29.0 | 25.9(4), 3.9(1) |
| 52  53 | HSD17B1  HSD17B1 | P14061  P14061 | Estradiol 17-β-dehydrogenase 1  Estradiol 17-β-dehydrogenase 1 | 328  328 | 4-312  23-328 | 671.0, 149.0  682.0, 118.0 | 49.2(16), 30.6(7)  64.6(22), 29.0(6) |
| 17 | HSPA5 | P11021 | 78 kDa glucose-regulated protein | 654 | 61-573 | 285.0, 80.0 | 35.0(17), 7.5(3) |
| 21 | HSP90B1 | P14625 | Endoplasmin | 803 | 76-690 | 149.0, 45.0 | 22.4(14), 1.4(1) |
| 1  2  3  4 | ITIH4  ITIH4  ITIH4  ITIH4 | Q14624  Q14624  Q14624  Q14624 | Inter-α-trypsin inhibitor heavy chain H4 (isoform 1)  Inter-α-trypsin inhibitor heavy chain H4 (isoform 1)  Inter-α-trypsin inhibitor heavy chain H4 (isoform 1)  Inter-α-trypsin inhibitor heavy chain H4 (isoform 1) | 930  930  930  930 | 48-777  48-777  48-733  2-733 | 267.0, 99.0  265.0, 116.0  260.0, 113.0  293.0, 116.0 | 24.1(19), 4.1(2)  17.8(13), 4.1(2)  14.4(8), 4.3(2)  19.7(12), 5.3(3) |
| 30  31  54*  57* | KRT8  KRT8  KRT8  KRT8 | P05787  P05787  P05787  P05787 | Keratin, type II cyto-skeletal 8 (isoform 1,2)  Keratin, type II cyto-skeletal 8 (isoform 1,2)  Keratin, type II cyto-skeletal 8 (isoform 1,2; fragment)  Keratin, type II cyto-skeletal 8 (isoform 1,2; fragment) | 483  483  483  483 | 24-454  24-414  134-414  134-401 | 845.0, 121.0  181.0, 57.0  857.0, 122.0  662.0, 113.0 | 60.2(32), 18.8(7)  38.5(17), 2.3(1)  66.8(30), 24.7(7)  58.4(26), 24.7(6) |
| 5  6 | KRT18  KRT18 | P05783  P05783 | Keratin, type I cytoskeletal 18  Keratin, type I cytoskeletal 18 | 430  430 | 7-426  7-426 | 292.0, 58.0  347.0, 61.0 | 51.6(21), 10.2(4)  53.8(23), 17.5(5) |
| 43  44  45  46  56* | KRT19  KRT19  KRT19  KRT19  KRT19 | P08727  P08727  P08727  P08727  P08727 | Keratin, type I cytoskeletal 19  Keratin, type I cytoskeletal 19  Keratin, type I cytoskeletal 19  Keratin, type I cytoskeletal 19  Keratin, type I cytoskeletal 19 (C-terminal fragment) | 400  400  400  400  400 | 8-381  8-381  8-381  8-398  91-398 | 809.0, 139.0  692.0, 127.0  740.0, 137.0  824.0, 143.0  590.0, 91.0 | 65.5(27), 18.0(6)  63.7(27), 18.0(6)  64.5(27), 16.8(5)  76.0(32), 20.0(6)  56.0(29), 21.3(9) |
| 33  34  35  36  37 | LUM  LUM  LUM  LUM  LUM | P51884  P51884  P51884  P51884  P51884 | Lumican  Lumican  Lumican  Lumican  Lumican | 338  338  338  338  338 | 70-330  70-338  70-338  70-330  70-338 | 235.0, 92.0  206.0, 88.0  229.0, 107.0  196.0, 83.0  210.0, 91.0 | 18.6(7), 10.9(3)  27.2(9), 6.5(2)  24.3(9), 6.5(2)  28.1(9), 6.5(2)  35.8(11), 6.5(2) |
| 79 | NUDT16 | Q96DE0 | U8 snoRNA-decapping enzyme (isoform 1) | 195 | 21-168 | 83.5, - | 47.7(7), - |
| 68 | PRDX4 | Q13162 | Peroxiredoxin-4 | 271 | 46-240 | 254.0, 63.0 | 51.6 (10), 15.0(3) |
| 75 | PRDX6 | P30041 | Peroxiredoxin-6 | 224 | 2-200 | 427.0, 69.0 | 70.5(16), 14.3(5) |
| 47 | SERPINB2 | P05120 | Plasminogen activator inhibitor 2 | 415 | 2-411 | 443.0, 134.0 | 43.4(18), 17.8(4) |
| 88 | S100A6 | P06703 | Protein S100-A6 | 90 | 33-64 | 136.0, 49.0 | 35.6(6), 16.7(2) |
| 80 | TAGLN2 | P37802 | Transgelin-2 (isoform 1,2) | 199 | 5-187 | 229.0, 73.0 | 50.3(10), 13.9(2) |
| 10 | TF | P02787 | Serotransferrin | 698 | 61-668 | 187.0, - | 30.1(22),- |
| 61  62 | TPM1  TPM1 | P09493  P09493 | Tropomyosin α-1 chain (isoform 2,3,4)  Tropomyosin α-1 chain (isoform 1) | 228  284 | 21-188  36-264 | 190.0, 270.4  273.0, 56.0 | 37.4(13), 31.4(11)  47.0(19), 9.5(2) |
| 55 | TPM2 | P07951 | Tropomyosin ß chain (isoform 2) | 284 | 13-284 | 618.0, 77.0 | 63.0(29), 11.3(5) |
| 41 | VIM | P08670 | Vimentin | 466 | 37-440 | 421.0, 129.0 | 43.1(17, 10.1(2) |
| 29 | WARS | P23381 | Tryptophan-tRNA ligase, cytoplasmic (isoform 1) | 471 | 2-471 | 553.0, 86.0 | 62.8(30), 14.4(6) |

Parameters of protein identification, including MS and MSMS Mascot scores as well as sequence coverage (SC) and covered peptides (P). Total number of amino acids and sequence covered by MS are also indicated. If the identified spot allowed for the presence of several isoforms, amino acids and sequence coverage are indicated for the most common isoform. If both spot position and sequence coverage indicate the presence of a protein fragment, it is marked by an asterisk.

**Table S4. Upregulated genes after *SERPINA1* silencing in HTR8/SVneo cells.**

| Gene name | EntrezID | *p* value^a^ | adj. *p* value^b^ | FC^c^ |
| --- | --- | --- | --- | --- |
| *ACTG2* | 72 | 0.00001 | 0.00000 | 10.93 |
| *EDN1* | 1906 | 0.00001 | 0.00000 | 7.33 |
| *MYL7* | 58498 | 0.00003 | 0.00000 | 5.16 |
| *TAGLN* | 6876 | 0.00005 | 0.00000 | 3.77 |
| *ACTC1* | 70 | 0.00012 | 0.00000 | 3.43 |
| *GBP1* | 2633 | 0.00004 | 0.00000 | 3.40 |
| *SYNPO2* | 171024 | 0.00008 | 0.00000 | 3.36 |
| *B3GALT2* | 8707 | 0.00021 | 0.00000 | 3.25 |
| *LPP* | 4026 | 0.00020 | 0.00000 | 3.00 |
| *ANKRD1* | 27063 | 0.00008 | 0.00000 | 2.98 |
| *GLIPR1* | 11010 | 0.00010 | 0.00000 | 2.90 |
| *SORBS2* | 8470 | 0.00016 | 0.00000 | 2.80 |
| *RGS4* | 5999 | 0.00023 | 0.00000 | 2.76 |
| *CXADR* | 1525 | 0.00018 | 0.00000 | 2.70 |
| *THBS1* | 7057 | 0.00015 | 0.00000 | 2.69 |
| *ARHGAP20* | 57569 | 0.00012 | 0.00000 | 2.62 |
| *SH3RF2* | 153769 | 0.00029 | 0.00000 | 2.51 |
| *ARHGDIB* | 397 | 0.00019 | 0.00000 | 2.46 |
| *EDIL3* | 10085 | 0.00025 | 0.00000 | 2.42 |
| *TGFB2* | 7042 | 0.00031 | 0.00000 | 2.39 |
| *GADD45B* | 4616 | 0.00032 | 0.00000 | 2.32 |
| *MBNL1-AS1* | 401093 | 0.00035 | 0.00000 | 2.31 |
| *SLIT3* | 6586 | 0.00057 | 0.00000 | 2.28 |
| *FGF1* | 2246 | 0.00061 | 0.00000 | 2.24 |
| *TENM2* | 57451 | 0.00058 | 0.00000 | 2.24 |
| *LCP1* | 3936 | 0.00040 | 0.00000 | 2.24 |
| *PLCB4* | 5332 | 0.00043 | 0.00000 | 2.22 |
| *CCN2* | 1490 | 0.00033 | 0.00000 | 2.22 |
| *S1PR1* | 1901 | 0.00036 | 0.00000 | 2.21 |
| *COL8A1* | 1295 | 0.00027 | 0.00000 | 2.20 |
| *TNC* | 3371 | 0.00054 | 0.00000 | 2.19 |
| *CALD1* | 800 | 0.00040 | 0.00000 | 2.19 |
| *GJA5* | 2702 | 0.00038 | 0.00000 | 2.18 |
| *PAWR* | 5074 | 0.00066 | 0.00000 | 2.17 |
| *ZC3HAV1L* | 92092 | 0.00081 | 0.00000 | 2.17 |
| *EFR3A* | 23167 | 0.00063 | 0.00000 | 2.15 |
| *RASGRP3* | 25780 | 0.00093 | 0.00000 | 2.15 |
| *SETD7* | 80854 | 0.00068 | 0.00000 | 2.14 |
| *LCLAT1* | 253558 | 0.00047 | 0.00000 | 2.10 |
| *FSTL1* | 11167 | 0.00044 | 0.00000 | 2.09 |
| *COL4A1* | 1282 | 0.00048 | 0.00000 | 2.09 |
| *NFIB* | 4781 | 0.00098 | 0.00000 | 2.08 |
| *SLIT2* | 9353 | 0.00087 | 0.00000 | 2.08 |
| *RECK* | 8434 | 0.00072 | 0.00000 | 2.06 |
| *FBXL17* | 64839 | 0.00072 | 0.00000 | 2.05 |
| *OSTM1* | 28962 | 0.00086 | 0.00000 | 2.05 |
| *PDCD4-AS1* | 282997 | 0.00065 | 0.00000 | 2.04 |
| *SLC26A4* | 5172 | 0.00077 | 0.00000 | 2.04 |
| *CCN1* | 3491 | 0.00060 | 0.00000 | 2.03 |
| *VGLL3* | 389136 | 0.00143 | 0.00000 | 2.02 |
| *MET* | 4233 | 0.00085 | 0.00000 | 2.02 |
| *LOC100506990* | 100506990 | 0.00076 | 0.00000 | 2.02 |
| *CADM1* | 23705 | 0.00102 | 0.00000 | 2.02 |
| *IGIP* | 492311 | 0.00124 | 0.00000 | 2.02 |
| *ZEB1-AS1* | 220930 | 0.00070 | 0.00000 | 2.02 |
| *MSRB3* | 253827 | 0.00073 | 0.00000 | 2.01 |
| *CD274* | 29126 | 0.00082 | 0.00000 | 2.00 |

*SERPINA1* was silenced in HTR8/SVneo commercial cell line by siRNA. Transcriptome of these cells was compared with transcriptome of cells treated with negative siRNA. Differentially expressed genes were ranked based on false discovery rate (FDR)–adjusted *p* value and fold change (FC). Threshold of FC was >2, and threshold of FDR-adjusted *p* value was <0.05.

^a^*t*-test *p* value for comparisons between sample groups (*SERPINA1*-silenced and negative control cells).

^b^FDR-adjusted *p* value.

^c^Expression ratio (FC) between compared sample groups. Comparison between *SERPINA1-*silenced cells and negative control cells.

**Table S5. Downregulated genes after *SERPINA1* silencing in HTR8/SVneo cells.**

| Gene name | EntrezID | *p* value^a^ | adj. *p* value^b^ | FC^c^ |
| --- | --- | --- | --- | --- |
| *GDF15* | 9518 | 0.00002 | 0.00000 | -4.85 |
| *INPP5D* | 3635 | 0.00003 | 0.00000 | -4.25 |
| *ALDH3B1* | 221 | 0.00006 | 0.00000 | -3.09 |
| *CEACAM1* | 634 | 0.00005 | 0.00000 | -3.02 |
| *CDKN1A* | 1026 | 0.00009 | 0.00000 | -2.96 |
| *TP53I3* | 9540 | 0.00014 | 0.00000 | -2.94 |
| *CD68* | 968 | 0.00011 | 0.00000 | -2.91 |
| *NECTIN4* | 81607 | 0.00010 | 0.00000 | -2.89 |
| *TOMM34* | 10953 | 0.00007 | 0.00000 | -2.86 |
| *CYGB* | 114757 | 0.00016 | 0.00000 | -2.83 |
| *CXCL8* | 3576 | 0.00013 | 0.00000 | -2.73 |
| *H2AC19* | 723790 | 0.00023 | 0.00000 | -2.72 |
| *FER1L4* | 80307 | 0.00026 | 0.00000 | -2.68 |
| *RASSF3* | 283349 | 0.00014 | 0.00000 | -2.66 |
| *CFI* | 3426 | 0.00018 | 0.00000 | -2.64 |
| *ST6GALNAC2* | 10610 | 0.00038 | 0.00000 | -2.52 |
| *IL1B* | 3553 | 0.00028 | 0.00000 | -2.50 |
| *PTGES* | 9536 | 0.00020 | 0.00000 | -2.50 |
| *IL1R1* | 3554 | 0.00025 | 0.00000 | -2.49 |
| *KANK3* | 256949 | 0.00030 | 0.00000 | -2.45 |
| *SERPINA1* | 5265 | 0.00017 | 0.00000 | -2.42 |
| *CLCA2* | 9635 | 0.00033 | 0.00000 | -2.41 |
| *FAM3C* | 10447 | 0.00031 | 0.00000 | -2.39 |
| *TTC1* | 7265 | 0.00022 | 0.00000 | -2.39 |
| *PROM2* | 150696 | 0.00024 | 0.00000 | -2.39 |
| *CXCL6* | 6372 | 0.00027 | 0.00000 | -2.37 |
| *NDRG4* | 65009 | 0.00029 | 0.00000 | -2.31 |
| *H1-2* | 3006 | 0.00039 | 0.00000 | -2.29 |
| *ITGAX* | 3687 | 0.00042 | 0.00000 | -2.25 |
| *BDKRB2* | 624 | 0.00055 | 0.00000 | -2.25 |
| *H2BC12* | 85236 | 0.00034 | 0.00000 | -2.24 |
| *CMBL* | 134147 | 0.00035 | 0.00000 | -2.23 |
| *NECAP1* | 25977 | 0.00037 | 0.00000 | -2.21 |
| *NCEH1* | 57552 | 0.00042 | 0.00000 | -2.21 |
| *KLHDC2* | 23588 | 0.00049 | 0.00000 | -2.18 |
| *TUBB3* | 10381 | 0.00041 | 0.00000 | -2.17 |
| *NMB* | 4828 | 0.00055 | 0.00000 | -2.17 |
| *LOC102724334* | 102724334 | 0.00066 | 0.00000 | -2.16 |
| *RCOR2* | 283248 | 0.00044 | 0.00000 | -2.16 |
| *SFRP1* | 6422 | 0.00050 | 0.00000 | -2.15 |
| *ADRA1D* | 146 | 0.00061 | 0.00000 | -2.14 |
| *CXCL1* | 2919 | 0.00052 | 0.00000 | -2.14 |
| *TDG* | 6996 | 0.00053 | 0.00000 | -2.14 |
| *BST2* | 684 | 0.00051 | 0.00000 | -2.13 |
| *SDSL* | 113675 | 0.00074 | 0.00000 | -2.12 |
| *PANX2* | 56666 | 0.00059 | 0.00000 | -2.12 |
| *HOXA1* | 3198 | 0.00089 | 0.00000 | -2.11 |
| *MELTF* | 4241 | 0.00046 | 0.00000 | -2.11 |
| *GRIN2C* | 2905 | 0.00113 | 0.00000 | -2.10 |
| *MYO15B* | 80022 | 0.00080 | 0.00000 | -2.10 |
| *H2AC6* | 8334 | 0.00048 | 0.00000 | -2.10 |
| *SULF2* | 55959 | 0.00056 | 0.00000 | -2.10 |
| *TSPAN10* | 83882 | 0.00078 | 0.00000 | -2.09 |
| *MVP* | 9961 | 0.00063 | 0.00000 | -2.07 |
| *PRAF2* | 11230 | 0.00057 | 0.00000 | -2.07 |
| *SERPINF1* | 5176 | 0.00069 | 0.00000 | -2.07 |
| *CLMP* | 79827 | 0.00100 | 0.00000 | -2.06 |
| *CDH10* | 1008 | 0.00068 | 0.00000 | -2.06 |
| *DQX1* | 165545 | 0.00100 | 0.00000 | -2.06 |
| *CEMIP* | 57214 | 0.00070 | 0.00000 | -2.05 |
| *GRAMD2A* | 196996 | 0.00053 | 0.00000 | -2.05 |
| *PROCR* | 10544 | 0.00046 | 0.00000 | -2.05 |
| *TNFRSF9* | 3604 | 0.00123 | 0.00000 | -2.05 |
| *TM7SF2* | 7108 | 0.00083 | 0.00000 | -2.05 |
| *CCDC85B* | 11007 | 0.00287 | 0.00000 | -2.05 |
| *KCNN1* | 3780 | 0.00085 | 0.00000 | -2.05 |
| *LOC283335* | 283335 | 0.00098 | 0.00000 | -2.04 |
| *ISG20* | 3669 | 0.00075 | 0.00000 | -2.03 |
| *DPY30* | 84661 | 0.00045 | 0.00000 | -2.02 |
| *IQCN* | 80726 | 0.00119 | 0.00000 | -2.02 |
| *SLC25A10* | 1468 | 0.00093 | 0.00000 | -2.02 |
| *TMEM230* | 29058 | 0.00059 | 0.00000 | -2.02 |
| *GNG7* | 2788 | 0.00091 | 0.00000 | -2.01 |
| *YJEFN3* | 374887 | 0.00096 | 0.00000 | -2.01 |
| *SFXN2* | 118980 | 0.00064 | 0.00000 | -2.00 |
| *MRFAP1* | 93621 | 0.00062 | 0.00000 | -2.00 |

*SERPINA1* was silenced in HTR8/SVneo commercial cell line by siRNA. Transcriptome of these cells was compared with transcriptome of cells treated with negative siRNA. Differentially expressed genes were ranked based on FDR-adjusted *p* value and fold change (FC). Threshold of FC was >2, and threshold of FDR-adjusted *p* value was <0.05.

^a^*t*-test *p* value for comparisons between sample groups (*SERPINA1*-silenced and negative control cells).

^b^FDR-adjusted *p* value.

^c^Expression ratio (FC) between compared sample groups. Comparison between *SERPINA1-*silenced cells and negative control cells.

**Table S6. Biological pathways affected by *SERPINA1* silencing in HTR8/SVneo cells**.

| Term^a^ | Count | % | *p* value^b^ | Benjamini^c^ |
| --- | --- | --- | --- | --- |
| Regulation of actin cytoskeleton | 26 | 2.04 | 1.3E-4 | 1.3E-2 |
| Amoebiasis | 17 | 1.06 | 1.4E-4 | 1.3E-2 |
| Pathways in cancer | 39 | 3.07 | 2.4E-4 | 1.3E-2 |
| Focal adhesion | 25 | 2.04 | 2.4E-4 | 1.3E-2 |
| FoxO signaling pathway | 19 | 1.08 | 2.6E-4 | 1.3E-2 |
| Chagas disease (American trypanosomiasis) | 15 | 1.04 | 1.2E-3 | 5.1E-2 |
| TNF signaling pathway | 15 | 1.04 | 1.6E-3 | 5.7E-2 |
| Hepatitis B | 18 | 1.07 | 1.8E-3 | 5.7E-2 |
| MAPK signaling pathway | 26 | 2.04 | 2.1E-3 | 5.7E-2 |
| Proteoglycans in cancer | 22 | 2.01 | 2.2E-3 | 5.7E-2 |
| Malaria | 9 | 0.08 | 4.1E-3 | 8.7E-2 |
| Osteoclast differentiation | 16 | 1.05 | 4.1E-3 | 8.7E-2 |
| Cytokine-cytokine receptor interaction | 24 | 2.03 | 5.3E-3 | 9.8E-2 |
| PI3K-Akt signaling pathway | 31 | 2.09 | 5.4E-3 | 9.8E-2 |
| Inflammatory mediator regulation of TRP channels | 13 | 1.02 | 5.8E-3 | 9.8E-2 |
| Axon guidance | 15 | 1.04 | 7.7E-3 | 1.2E-1 |
| Leukocyte transendothelial migration | 14 | 1.03 | 8.1E-3 | 1.2E-1 |
| HTLV-I infection | 24 | 2.03 | 8.9E-3 | 1.3E-1 |
| Complement and coagulation cascades | 10 | 0.09 | 1.1E-2 | 1.4E-1 |
| Insulin resistance | 13 | 1.02 | 1.2E-2 | 1.5E-1 |
| Salmonella infection | 11 | 1.00 | 1.3E-2 | 1.5E-1 |
| Melanoma | 10 | 0.09 | 1.3E-2 | 1.5E-1 |
| Biosynthesis of amino acids | 10 | 0.09 | 1.4E-2 | 1.5E-1 |
| Cysteine and methionine metabolism | 7 | 0.07 | 1.5E-2 | 1.5E-1 |
| Glycine, serine and threonine metabolism | 7 | 0.07 | 1.6E-2 | 1.5E-1 |
| Colorectal cancer | 9 | 0.08 | 1.7E-2 | 1.5E-1 |
| Rap1 signaling pathway | 20 | 1.09 | 1.7E-2 | 1.5E-1 |
| NF-kappa B signaling pathway | 11 | 1.00 | 1.7E-2 | 1.5E-1 |
| Hematopoietic cell lineage | 11 | 1.00 | 1.7E-2 | 1.5E-1 |
| Pertussis | 10 | 0.09 | 1.8E-2 | 1.5E-1 |
| Bacterial invasion of epithelial cells | 10 | 0.09 | 2.3E-2 | 1.9E-1 |
| p53 signaling pathway | 9 | 0.08 | 2.6E-2 | 2.0E-1 |
| Sphingolipid signaling pathway | 13 | 1.02 | 2.6E-2 | 2.0E-1 |
| Other types of O-glycan biosynthesis | 5 | 0.05 | 2.7E-2 | 2.0E-1 |
| Renin-angiotensin system | 5 | 0.05 | 3.2E-2 | 2.3E-1 |
| Metabolic pathways | 79 | 7.04 | 4.5E-2 | 2.9E-1 |
| Rheumatoid arthritis | 10 | 0.09 | 4.5E-2 | 2.9E-1 |
| Prostate cancer | 10 | 0.09 | 4.5E-2 | 2.9E-1 |
| Platelet activation | 13 | 1.02 | 4.5E-2 | 2.9E-1 |
| Apoptosis | 8 | 0.08 | 4.6E-2 | 2.9E-1 |

*SERPINA1* was silenced in HTR8/SVneo commercial cell line by siRNA. Transcriptome of these cells was compared with transcriptome of cells treated with negative siRNA. KEGG pathways were ranked based on *p* value. Threshold of *p* value was <0.05.

^a^Functional analysis of differentially expressed genes was conducted against KEGG database.

^b^Pathways with *p* < 0.05 are shown.

^c^Benjamini-Hochberg correction

**Table S7. Gene Ontology Biological Processes (GO-BP) term enrichment analysis of genes affected by *SERPINA1* silencing.**

| Term^a^ | Count | % | *p* value^b^ | Benjamini^c^ |
| --- | --- | --- | --- | --- |
| positive regulation of cell migration | 27 | 2.05 | 1.8E-6 | 6.1E-3 |
| extracellular matrix organization | 27 | 2.05 | 5.8E-6 | 1.0E-2 |
| positive regulation of gene expression | 30 | 2.08 | 5.7E-5 | 6.6E-2 |
| response to wounding | 12 | 1.01 | 2.7E-4 | 2.3E-1 |
| positive regulation of endothelial cell proliferation | 12 | 1.01 | 6.1E-4 | 3.7E-1 |
| positive regulation of angiogenesis | 16 | 1.05 | 6.4E-4 | 3.7E-1 |
| positive regulation of neuron projection development | 13 | 1.02 | 1.6E-3 | 7.7E-1 |
| positive regulation of release of sequestered calcium ion into cytosol | 7 | 0.07 | 1.9E-3 | 7.7E-1 |
| negative regulation of protein binding | 10 | 0.09 | 2.0E-3 | 7.7E-1 |
| protein O-linked fucosylation | 5 | 0.05 | 2.3E-3 | 7.8E-1 |
| response to mechanical stimulus | 10 | 0.09 | 2.6E-3 | 8.0E-1 |
| inflammatory response | 33 | 3.01 | 2.9E-3 | 8.4E-1 |
| cell adhesion | 38 | 3.06 | 3.2E-3 | 8.6E-1 |
| angiogenesis | 22 | 2.01 | 4.3E-3 | 9.9E-1 |
| regulation of cytosolic calcium ion concentration | 7 | 0.07 | 4.7E-3 | 9.9E-1 |
| negative regulation of cell-matrix adhesion | 5 | 0.05 | 5.5E-3 | 9.9E-1 |
| response to peptide hormone | 8 | 0.08 | 6.0E-3 | 9.9E-1 |
| response to hypoxia | 18 | 1.07 | 6.0E-3 | 9.9E-1 |
| cytokine-mediated signaling pathway | 15 | 1.04 | 6.2E-3 | 9.9E-1 |
| in utero embryonic development | 19 | 1.08 | 6.3E-3 | 9.9E-1 |
| negative chemotaxis | 7 | 0.07 | 6.4E-3 | 9.9E-1 |
| axon guidance | 17 | 1.06 | 6.4E-3 | 9.9E-1 |
| wound healing | 11 | 1.00 | 6.6E-3 | 9.9E-1 |
| muscle contraction | 13 | 1.02 | 7.4E-3 | 1.0E0 |
| positive regulation of cytosolic calcium ion concentration | 15 | 1.04 | 7.5E-3 | 1.0E0 |
| activation of MAPKK activity | 8 | 0.08 | 7.6E-3 | 1.0E0 |
| negative regulation of cell growth | 14 | 1.03 | 7.8E-3 | 1.0E0 |
| leukocyte migration | 14 | 1.03 | 8.3E-3 | 1.0E0 |
| protein targeting to plasma membrane | 6 | 0.06 | 8.6E-3 | 1.0E0 |
| negative regulation of T cell receptor signaling pathway | 5 | 0.05 | 8.9E-3 | 1.0E0 |
| cellular response to tumor necrosis factor | 13 | 1.02 | 9.2E-3 | 1.0E0 |
| oxidation-reduction process | 44 | 4.01 | 1.0E-2 | 1.0E0 |
| positive regulation of vascular endothelial growth factor production | 6 | 0.06 | 1.0E-2 | 1.0E0 |
| integrin-mediated signaling pathway | 12 | 1.01 | 1.1E-2 | 1.0E0 |
| kidney development | 11 | 1.00 | 1.1E-2 | 1.0E0 |
| negative regulation of hormone secretion | 4 | 0.04 | 1.2E-2 | 1.0E0 |
| positive regulation of GTPase activity | 42 | 4.00 | 1.2E-2 | 1.0E0 |
| positive regulation of cell proliferation | 36 | 3.04 | 1.2E-2 | 1.0E0 |
| lung development | 10 | 0.09 | 1.4E-2 | 1.0E0 |
| negative regulation of endothelial cell proliferation | 6 | 0.06 | 1.4E-2 | 1.0E0 |
| homeostasis of number of cells within a tissue | 6 | 0.06 | 1.4E-2 | 1.0E0 |
| hydrogen sulfide biosynthetic process | 3 | 0.03 | 1.4E-2 | 1.0E0 |
| type I interferon signaling pathway | 9 | 0.08 | 1.4E-2 | 1.0E0 |
| positive regulation of macrophage chemotaxis | 4 | 0.04 | 1.5E-2 | 1.0E0 |
| creatine metabolic process | 4 | 0.04 | 1.5E-2 | 1.0E0 |
| atrioventricular valve morphogenesis | 4 | 0.04 | 1.5E-2 | 1.0E0 |
| positive regulation of I-kappaB kinase/NF-kappaB signaling | 16 | 1.05 | 1.6E-2 | 1.0E0 |
| face morphogenesis | 6 | 0.06 | 1.6E-2 | 1.0E0 |
| anatomical structure morphogenesis | 11 | 1.00 | 1.7E-2 | 1.0E0 |
| regulation of small GTPase mediated signal transduction | 14 | 1.03 | 1.7E-2 | 1.0E0 |
| response to amino acid | 6 | 0.06 | 1.8E-2 | 1.0E0 |
| response to lipopolysaccharide | 16 | 1.05 | 1.8E-2 | 1.0E0 |
| positive regulation of smooth muscle contraction | 5 | 0.05 | 1.9E-2 | 1.0E0 |
| cellular response to gamma radiation | 5 | 0.05 | 1.9E-2 | 1.0E0 |
| defense response to virus | 16 | 1.05 | 1.9E-2 | 1.0E0 |
| negative regulation of gene expression | 14 | 1.03 | 2.1E-2 | 1.0E0 |
| positive regulation of vasoconstriction | 6 | 0.06 | 2.1E-2 | 1.0E0 |
| negative regulation of cell migration | 11 | 1.00 | 2.1E-2 | 1.0E0 |
| transmembrane transport | 21 | 2.00 | 2.2E-2 | 1.0E0 |
| heart development | 17 | 1.06 | 2.2E-2 | 1.0E0 |
| transsulfuration | 3 | 0.03 | 2.3E-2 | 1.0E0 |
| positive regulation of mucus secretion | 3 | 0.03 | 2.3E-2 | 1.0E0 |
| protein localization to basolateral plasma membrane | 3 | 0.03 | 2.3E-2 | 1.0E0 |
| negative regulation of mitochondrial depolarization | 3 | 0.03 | 2.3E-2 | 1.0E0 |
| cellular response to heparin | 3 | 0.03 | 2.3E-2 | 1.0E0 |
| mesenchyme migration | 3 | 0.03 | 2.3E-2 | 1.0E0 |
| regulation of fatty acid metabolic process | 3 | 0.03 | 2.3E-2 | 1.0E0 |
| cellular response to growth factor stimulus | 7 | 0.07 | 2.4E-2 | 1.0E0 |
| response to arsenic-containing substance | 4 | 0.04 | 2.5E-2 | 1.0E0 |
| digestive tract morphogenesis | 4 | 0.04 | 2.5E-2 | 1.0E0 |
| atrial septum morphogenesis | 4 | 0.04 | 2.5E-2 | 1.0E0 |
| protein oligomerization | 8 | 0.08 | 2.6E-2 | 1.0E0 |
| response to toxic substance | 10 | 0.09 | 2.7E-2 | 1.0E0 |
| positive regulation of endothelial cell migration | 7 | 0.07 | 2.7E-2 | 1.0E0 |
| negative regulation of cell proliferation | 30 | 2.08 | 2.7E-2 | 1.0E0 |
| movement of cell or subcellular component | 10 | 0.09 | 2.9E-2 | 1.0E0 |
| positive regulation of apoptotic process | 24 | 2.03 | 2.9E-2 | 1.0E0 |
| cell proliferation | 28 | 2.06 | 3.0E-2 | 1.0E0 |
| O-glycan processing | 8 | 0.08 | 3.0E-2 | 1.0E0 |
| regulation of calcium ion transport | 5 | 0.05 | 3.0E-2 | 1.0E0 |
| uterus development | 4 | 0.04 | 3.0E-2 | 1.0E0 |
| glial cell differentiation | 4 | 0.04 | 3.0E-2 | 1.0E0 |
| nucleoside triphosphate biosynthetic process | 4 | 0.04 | 3.0E-2 | 1.0E0 |
| venous blood vessel morphogenesis | 3 | 0.03 | 3.3E-2 | 1.0E0 |
| maternal process involved in parturition | 3 | 0.03 | 3.3E-2 | 1.0E0 |
| endothelium development | 3 | 0.03 | 3.3E-2 | 1.0E0 |
| response to peptidoglycan | 3 | 0.03 | 3.3E-2 | 1.0E0 |
| negative regulation of neuron apoptotic process | 13 | 1.02 | 3.4E-2 | 1.0E0 |
| regulation of ERK1 and ERK2 cascade | 5 | 0.05 | 3.5E-2 | 1.0E0 |
| cellular response to transforming growth factor beta stimulus | 7 | 0.07 | 3.5E-2 | 1.0E0 |
| DNA damage response, signal transduction by p53 class mediator resulting in cell cycle arrest | 8 | 0.08 | 3.5E-2 | 1.0E0 |
| negative regulation of angiogenesis | 8 | 0.08 | 3.5E-2 | 1.0E0 |
| negative regulation of cell adhesion | 6 | 0.06 | 3.6E-2 | 1.0E0 |
| calcium ion transport | 9 | 0.08 | 3.7E-2 | 1.0E0 |
| endoplasmic reticulum calcium ion homeostasis | 4 | 0.04 | 3.7E-2 | 1.0E0 |
| neuromuscular process | 4 | 0.04 | 3.7E-2 | 1.0E0 |
| positive regulation of sequence-specific DNA binding transcription factor activity | 11 | 1.00 | 3.8E-2 | 1.0E0 |
| positive regulation of mitotic nuclear division | 5 | 0.05 | 3.9E-2 | 1.0E0 |
| positive regulation of cell-substrate adhesion | 6 | 0.06 | 4.0E-2 | 1.0E0 |
| collagen catabolic process | 8 | 0.08 | 4.1E-2 | 1.0E0 |
| regulation of phosphatidylinositol 3-kinase signaling | 9 | 0.08 | 4.2E-2 | 1.0E0 |
| chemotaxis | 12 | 1.01 | 4.3E-2 | 1.0E0 |
| cell chemotaxis | 8 | 0.08 | 4.4E-2 | 1.0E0 |
| response to progesterone | 6 | 0.06 | 4.4E-2 | 1.0E0 |
| homocysteine metabolic process | 3 | 0.03 | 4.5E-2 | 1.0E0 |
| response to dexamethasone | 3 | 0.03 | 4.5E-2 | 1.0E0 |
| response to cytokine | 7 | 0.07 | 4.5E-2 | 1.0E0 |

*SERPINA1* was silenced in HTR8/SVneo commercial cell line by siRNA. Transcriptome of these cells was compared with transcriptome of cells treated with negative siRNA. GO terms were ranked based on *p* value. Threshold of *p* value was <0.05.

^a^Functional analysis of differentially expressed genes was conducted against GO database.

^b^Gene ontologies with *p* < 0.05 are shown.

^c^Benjamini-Hochberg correction

**Table S8. Gene Ontology Cellular Component (GO-CC) term enrichment analysis of genes affected by *SERPINA1* silencing.**

| Term^a^ | Count | % | P-Value^b^ | Benjamini^c^ |
| --- | --- | --- | --- | --- |
| proteinaceous extracellular matrix | 32 | 3.00 | 1.9E-5 | 9.9E-3 |
| extracellular exosome | 186 | 17.05 | 1.1E-4 | 2.1E-2 |
| cell periphery | 10 | 0.09 | 1.2E-4 | 2.1E-2 |
| basement membrane | 14 | 1.03 | 1.7E-4 | 2.2E-2 |
| cell surface | 48 | 4.05 | 2.9E-4 | 3.0E-2 |
| extracellular space | 98 | 9.02 | 3.5E-4 | 3.0E-2 |
| endoplasmic reticulum membrane | 66 | 6.02 | 1.1E-3 | 8.0E-2 |
| endoplasmic reticulum lumen | 21 | 2.00 | 2.0E-3 | 1.2E-1 |
| membrane raft | 22 | 2.01 | 2.1E-3 | 1.2E-1 |
| extracellular matrix | 28 | 2.06 | 2.7E-3 | 1.3E-1 |
| Golgi membrane | 47 | 4.04 | 3.1E-3 | 1.3E-1 |
| cell-cell junction | 19 | 1.08 | 3.1E-3 | 1.3E-1 |
| interstitial matrix | 5 | 0.05 | 3.4E-3 | 1.3E-1 |
| cytoplasmic vesicle | 23 | 2.02 | 4.7E-3 | 1.7E-1 |
| basal lamina | 5 | 0.05 | 5.9E-3 | 2.0E-1 |
| focal adhesion | 33 | 3.01 | 6.1E-3 | 2.0E-1 |
| actin cytoskeleton | 21 | 2.00 | 8.4E-3 | 2.6E-1 |
| extracellular region | 104 | 9.08 | 1.0E-2 | 2.9E-1 |
| cytoplasmic, membrane-bounded vesicle | 15 | 1.04 | 1.1E-2 | 3.0E-1 |
| cell junction | 36 | 3.04 | 1.2E-2 | 3.1E-1 |
| myofibril | 6 | 0.06 | 1.3E-2 | 3.1E-1 |
| cytosol | 197 | 18.05 | 1.3E-2 | 3.1E-1 |
| basal plasma membrane | 6 | 0.06 | 1.7E-2 | 3.8E-1 |
| neuron projection | 21 | 2.00 | 2.0E-2 | 4.2E-1 |
| nuclear inner membrane | 7 | 0.07 | 2.1E-2 | 4.4E-1 |
| perinuclear region of cytoplasm | 44 | 4.01 | 2.7E-2 | 5.0E-1 |
| postsynaptic density | 17 | 1.06 | 2.7E-2 | 5.0E-1 |
| filopodium | 9 | 0.08 | 2.8E-2 | 5.0E-1 |
| sarcoplasmic reticulum | 6 | 0.06 | 2.8E-2 | 5.0E-1 |
| basolateral plasma membrane | 16 | 1.05 | 4.3E-2 | 7.4E-1 |
| rough endoplasmic reticulum | 7 | 0.07 | 4.5E-2 | 7.5E-1 |

*SERPINA1* was silenced in HTR8/SVneo commercial cell line by siRNA. Transcriptome of these cells was compared with transcriptome of cells treated with negative siRNA. GO terms were ranked based on *p* value. Threshold of *p* value was <0.05.

^a^Functional analysis of differentially expressed genes was conducted against GO database.

^b^Gene Ontologies with *p* < 0.05 are shown.

^c^Benjamini-Hochberg correction

**Table S9.** Comparison of RNA sequencing and qRT-PCR of genes affected by *SERPINA1* knockdown.

| Gene ID | Description | RNA seq:  *SERPINA1* knockdown |  | qRT-PCR: *SERPINA1*  knockdown |  |
| --- | --- | --- | --- | --- | --- |
|  |  | ***p* - value^a^** | **FC^b^** | ***p* - value^c^** | **FC^b^** |
| *SERPINA1* | Serpin Family A Member 1 | 0.0002 | -2.42 | 0.004 | -29.67 |
| *ACTG2* | Actin Gamma 2, Smooth Muscle | 0.00001 | 10.93 | 0.004 | 16.41 |
| *CEACAM1* | Carcinoembryonic antigen-related cell adhesion molecule 1 | 0.0001 | -3.02 | 0.004 | -3.24 |
| *FN1* | Fibronectin 1 | 0.00003 | 1.83 | 0.004 | 2.81 |
| *SLIT2* | Slit Guidance Ligand 2 | 0.001 | 2.08 | 0.004 | 2.29 |

^a^*t*-test *p* values after multiple hypothesis correction for comparisons between sample groups (*SERPINA1*-silenced and negative control cells).

^b^Expression ratio (fold change) between compared sample groups. Comparison between *SERPINA1-*silenced cells and negative control cells.

^c^Mann–Whitney *U* test *p* value.
